# Effects of gender-transformative relationships and sexuality education to reduce adolescent pregnancy (the JACK trial): a cluster-randomised trial

**DOI:** 10.1016/S2468-2667(22)00117-7

**Published:** 2022-07-01

**Authors:** Maria Lohan, Aoibheann Brennan-Wilson, Rachael Hunter, Andrea Gabrio, Lisa McDaid, Honor Young, Rebecca French, Áine Aventin, Mike Clarke, Clíona McDowell, Danielle Logan, Sorcha Toase, Liam O’Hare, Chris Bonell, Katie Gillespie, Aisling Gough, Susan Lagdon, Emily Warren, Kelly Buckley, Ruth Lewis, Linda Adara, Theresa McShane, Julia Bailey, James White

**Affiliations:** School of Nursing and Midwifery; Queen’s University Belfast, Belfast, Northern Ireland, UK; Health Economics Analysis and Research Methods Team; University College London, London, England, UK; Department of Methodology and Statistics (FHML), Maastricht University, Maastricht, Netherlands; MRC–CSO Social and Public Health Sciences Unit, University of Glasgow, Glasgow, Scotland, UK; Institute for Social Science Research, The University of Queensland, Brisbane, QLD, Australia; Centre for Development, Evaluation, Complexity and Implementation in Public Health Improvement; Department of Public Health Environments and Society, London School of Hygiene and Tropical Medicine, London, England, UK; School of Nursing and Midwifery; School of Medicine, Dentistry and Biomedical Sciences; Cardiff University, Cardiff, Wales, UK; Northern Ireland Clinical Trials Unit, Belfast, Northern Ireland, UK; Cardiff University, Cardiff, Wales, UK; Northern Ireland Clinical Trials Unit, Belfast, Northern Ireland, UK; School of Social Sciences, Education and Social Work; Department of Public Health Environments and Society, London School of Hygiene and Tropical Medicine, London, England, UK; School of Social Sciences, Education and Social Work; School of Nursing and Midwifery; School of Psychology, Ulster University, Coleraine, Northern Ireland, UK; Department of Public Health Environments and Society, London School of Hygiene and Tropical Medicine, London, England, UK; Y Lab, School of Social Sciences; MRC–CSO Social and Public Health Sciences Unit, University of Glasgow, Glasgow, Scotland, UK; Centre for Trials Research; School of Psychology; E-Health Unit; Centre for Development, Evaluation, Complexity and Implementation in Public Health Improvement; Centre for Trials Research

## Abstract

**Background:**

The need to engage boys in gender-transformative relationships and sexuality education (RSE) to reduce adolescent pregnancy is endorsed by WHO. We aimed to test an intervention which used a gender-transformative approach to engage adolescents in RSE to prevent unprotected sex.

**Methods:**

This cluster-randomised trial with process and economic evaluations tested a school-based intervention entitled If I Were Jack versus standard RSE (control) for students (aged 14–15 years) in UK schools. Schools were randomly allocated (1:1) and masked to allocation at baseline. The primary outcome was self-reported avoidance of unprotected sex (sexual abstinence or use of reliable contraception at last sex) after 12–14-months. We analysed the data using intention-to-treat mixed effects regression models.

**Findings:**

Of 803 schools assessed for eligibility, 263 schools were invited by letter, of which 66 schools agreed to be randomly assigned, of which 62 schools completed follow-up. The trial was done between Feb 1, 2018, and March 6, 2020. 8216 students participated at baseline in 2018; 6561 (79·85%) provided 12-14 months follow-up. There was no significant difference in the primary outcome of avoidance of unprotected sex: 2648 (86·62) of 3057 in the intervention group avoided unprotected sex versus 2768 (86·41%) of 3203 in the control group (adjusted odds ratio [aOR] 0.85 [95% CI 0·58-1·26], p=0·42). Exploratory post-hoc analysis of the two components of the primary outcome showed that significantly more intervention students used reliable contraception at last sex compared with control students and there was no significant difference between the groups for sexual abstinence. No adverse events were reported.

**Interpretation:**

The intervention had a null effect on the primary outcome of preventing unprotected sex (increasing sexual abstinence or use of reliable contraception) in the whole student population. However, the results showed significant increases in use of reliable contraceptives for sexually active students. Engaging all young people early through RSE is important so that as they become sexually active, rates of unprotected sex are reduced.

**Funding:**

National Institute for Health Research.

## Introduction

The UK has the highest rate of adolescent pregnancy in western Europe.^1^ Adolescent unintended pregnancy is too often considered a women’s issue, both in how the problems are measured and in where solutions are sought.^2^ WHO and UNESCO^3^ among others have highlighted that greater engagement with men and boys through so-called gender-transformative relationships and sexuality education (RSE) that challenges gender inequalities is required to reduce unintended adolescent pregnancy and improve sexual and reproductive health and rights (SRHR) for all.^3–5^ School-based RSE during the adolescent years provides an efficient method of promoting gender equality and SRHR.^3,4,6–8^

We developed and piloted a school-based intervention entitled If I Were Jack in the UK for adolescents aged 14–15 years, which we designed using three of the most promising approaches to RSE. The first is a comprehensive approach to RSE. Unprotected sex and unintended pregnancy during adolescence are complex phenomena, which might not be prevented through RSE alone.^6,7,9–11^ However, high-quality, comprehensive, school-based RSE can equip children and adolescents with the knowledge to navigate reproductive health, sexual health, and sexuality issues at the right time.^6,7,11^ Systematic reviews and trial evidence suggest that comprehensive school-based RSE is more effective than an abstinence-only-until-marriage approach^12^ and more cost-effective than extracurricular interventions that are delivered outside of school.^13^ The If I Were Jack intervention is aimed at decreasing unintended pregnancy and increasing positive sexual health and relationships by encouraging adolescents to delay sexual activity until ready and to use effective contraception once sexually active (see JACK trial logic model, appendix p 3).

The second promising approach is the inclusion of effective RSE programme components on the basis of systematic review evidence,^6,9,11,14–16^ and information that responds to what young people say they want in RSE.^17^ Informed by these reviews, If I Were Jack addresses several psychosocial mediating variables associated with the use of reliable contraception, including knowledge about contraception, perceptions of norms about sexual behaviour and contraception, self-efficacy to communicate about sexual consent, and intentions to use reliable contraception.^6,11,14^ It also includes the use of culturally sensitive interactive digital modalities to promote personal identification and engagement;^15^ the use of skills-building components and opportunities for discussion and critical reflection;^11,14,17^ the involvement of parents;^11,16^ and facilitation of links with sexual and reproductive support services in each of the four nations of the UK.^9^

The third approach is to change RSE to promote greater engagement with boys and challenge gender inequalities. The need for greater male engagement and the need for a so-called gender-transformative approach, which explicitly challenges gender inequalities, has been highlighted by global health^4^ and education organisations^3^ as well as the European Society of Experts on Sex Education in their international technical guidance on RSE^18^ and identified as a gap in the most recent systematic review of reviews of RSE.^11^ RSE is often gender blind, assuming adolescent males and females will engage equally, and fails to challenge the gender inequalities in roles and responsibilities for safe sex that disproportionately lead to poorer SRHR for girls, but also place boys at risk.^2,4,5,8^ The If I Were Jack intervention invites adolescents to engage in adolescent boys’ perspectives while equally inviting adolescents to challenge gender inequalities associated with male sexual desire and female reproductive responsibility. The intervention promotes positive masculinities that generate gender equality in sexual and intimate relationships, and especially encourages boys to take an equal responsibility to girls in preventing adolescent pregnancy. To the best of our knowledge, there have been no randomised trials of male engagement gender-transformative school-based RSE addressing adolescent pregnancy.^19,20^

If I Were Jack incorporates these evidence-informed approaches to RSE and has been developed and refined through more than a decade of research with substantial input from young people, teachers, and RSE policy-makers.^21^ Findings from a pilot cluster-randomised trial in Northern Ireland in 2015 and a further pilot study in the remainder of the UK, following cultural adaptions, showed that the intervention was feasible to deliver and acceptable to participants.^22^ Here, we report the results of a phase 3 cluster-randomised trial with embedded economic and process evaluations of the If I Were Jack intervention. We hypothesised that schools using the intervention would have lower rates of self-reported unprotected sex compared with schools receiving standard RSE. We report the effectiveness and cost-effectiveness of the intervention on student outcomes at 12–14 months.

## Methods

### Study design and participants

We did a cluster-randomised trial, with process and economic evaluations, in 66 secondary schools across the four nations of the UK with schools as the unit of allocation.^21^ We included all students in the target year group at baseline (aged 14 years) with follow-up 12–14 months later (aged 15 years). The follow-up time was chosen to facilitate a 12–14 months follow-up of pupils (post-intervention) before some students exit formal education following their first major statutory exams or reaching the age of 16 years. There were no ineligibility criteria for students. We enrolled mainstream secondary schools within the state system, excluding schools involved in previous studies involving the intervention (n=17 schools) between February and September, 2018. A list of eligible schools was identified by accessing the statutory education school list websites in each of the UK nations. Schools with less than 30 students per target year group were excluded to enhance efficiency of data collection per school. School recruitment was stratified by nation and median school-level free school meal (FSM) entitlement. This is a widely used measure of school-level socioeconomic disadvantage and is assessed as the number of pupils per school eligible to receive free school meals on the basis of family income. The published protocol^21^ was amended during the trial to refine the methods. All amendments (appendix p 2) were approved by the independent Trial Steering Committee and completed before analysis. The trial was approved by a Queen’s University Belfast Research Ethics Committee on July 7, 2017. Written, informed consent for random allocation was obtained from a member of each school senior leadership team, and from students, teachers, and intervention delivery staff for data collection. Parents were also informed about the trial and offered the opportunity to withdraw their child from the trial (though not the intervention).

### Randomisation and masking

We used stratified block randomisation, with strata defined by the same school-level factors as random sampling. Schools were randomly ordered within each stratum. We randomly allocated schools to the intervention or control group (1:1) within each stratum. Sequence allocation was generated by an independent statistician from the Northern Ireland Clinical Trials Unit (NICTU) by means of random permuted blocks of mixed size, generated by means of nQuery Advisor 7.0. Schools were masked to allocation at baseline. After allocation was revealed, the intervention team, the process team, and the economic evaluation team were not masked to allocation status. However, fieldwork staff and staff who completed the data entry were masked to allocation throughout the trial.

### Procedures

Baseline questionnaires were administered in September-October, 2018. Delivery of the intervention began in October, 2018. Follow-up questionnaires were administered 12–14 months after baseline in November 2019-January, 2020. Student self-reported data were collected by means of paper-based questionnaires, which were completed by students under examination conditions in school, and facilitated by trained researchers. Students were assured of the confidentiality of their answers which was communicated in person and through a participant information sheet and information video. Fieldworkers only supported students requiring extra help and ensured questionnaires were completed confidentially. After data collection, questionnaires were reviewed for potential serious adverse events or disclosures that required safeguarding. Questionnaires were then scanned, and password protected scans sent to NICTU and stored on a secure server.

Both the intervention and trial methods first underwent extensive feasibility testing in a pilot, cluster-randomised trial in eight schools in Northern Ireland. We engaged a young persons’ advisory group and an RSE experts group across the UK to tailor the cultural relevance of the intervention to each of the UK nations and piloted the resource in a further nine schools in the UK. The development and optimisation of the If I Were Jack intervention is described in detail elsewhere.^21^ It is a brief intervention designed to be delivered by trained teachers during four or six consecutive RSE lessons in classroom settings (depending on normal class durations and scheduling). The intervention was designed to augment, rather than replace existing RSE in intervention schools. Schools allocated to the intervention group were provided with 90-min face-to-face training sessions for teachers; the If I Were Jack interactive video drama; classroom materials for teachers; online materials for parents or guardians; and information brochures and factsheets about adolescent pregnancy. Schools assigned to the control group continued with standard RSE lessons throughout their involvement in the trial and had no access to the intervention. See the Template for Intervention Description and Replication description for a detailed outline of the intervention (appendix pp 4-5).

We did a process evaluation to assess trial context, fidelity, and mechanisms of effect. For trial context, we examined reasons for school participation and non-participation, using correspondence with schools, interviews with school staff, and questionnaires. We also examined RSE delivered outside of If I Were Jack in intervention and control schools. Schools were categorised as having high, medium, or low provision of RSE assessed by means of a UK RSE quality assessment tool (appendix p 11). A fidelity checklist was used to assess fidelity of teacher training on the basis of a random selection of audio recordings. Classroom implementation was assessed by means of teacher implementation logs, interviews, and focus groups with teachers and students, and researcher observations in eight intervention schools (randomly selected by NICTU). We examined implementation of the parental component using teacher implementation logs and interviews, student questionnaires, and an online survey with parents and parent focus groups. Mechanisms of effect focused on participants’ perceptions of effectiveness and were assessed by means of a student questionnaire in all intervention schools, and interviews and focus groups with teachers and students in the eight case study schools (appendix pp 8–9).

We did a within-trial economic analysis to assess programme costs, on the basis of students’ health-care resource use (assessed by means of the trial questionnaire), and teacher resource use in the delivery of the intervention (assessed by means of a teacher resource use questionnaire). We also used a decision analytic model to report the long-term cost-effectiveness of the intervention. The model was populated by means of prespecified outcomes^21^ collected from the trial and published literature on the outcomes, which are directly and indirectly related to the distal outcomes of pregnancies and sexually transmitted infections (STIs) among adolescents (see appendix pp 14–18).

### Outcomes

**Figure 1:**
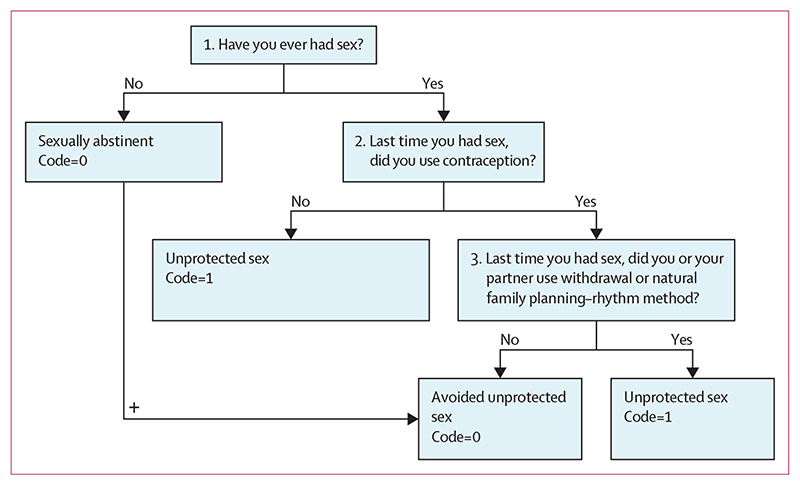
Flow diagram showing how the primary outcome was assessed 0=avoided unprotected sex. 1=unprotected sex.

The primary outcome was self-reported avoidance of unprotected sex (sexual-abstinence or use of reliable contraception at last sex) after 12–14 months. As the focus of the study was unintended pregnancy, sex was defined as penile–vaginal sex. A definition of this type of sex along with other terms was provided to students in questionnaires on the basis of definitions coconstructed and pilot tested with students in the feasibility trial.^23^ We used this surrogate measure associated with unintended adolescent pregnancy because the sample size would need to be very large to detect differences in pregnancy rates.^21^ Population-level data for the four nations of the UK reveal that between 25 and 33% of the population are sexually active by age 15 years and approximately 2·98% report having unprotected sex at last sex.^21^ Avoidance of unprotected sex was defined as either sexual abstinence or use of reliable contraception at last sex. Reliable contraception included hormonal or barrier methods. Unreliable contraception included the so-called with-drawal method and natural family planning–rhythm method. The questions used to derive the primary outcome are shown in figure 1. The selection of secondary outcomes was informed by our theory of change (appendix p 3). A full description and citations for the secondary outcomes are provided in the appendix (pp 6–7). The secondary outcomes included sexual health knowledge measured by means of items from the Mathtech Knowledge Inventory and SKATA; attitudes towards male gender roles, measured by means of the Male Role Attitudes Scale; skills, measured by means of the Comfort Communicating Scale, and the Sexual Self-Efficacy Scale; and, finally, intentions to avoid an unintended pregnancy measured by means of a so-called Intentions to Avoid a Teenage Pregnancy Scale. We also did an exploratory post-hoc analysis of the two components of the primary outcome.

### Statistical analysis

A statistical analysis plan was developed by NICTU and approved by the Trial Steering Committee defining the analyses of all primary and secondary outcomes and subgroups on the basis of the published protocol^21^ in advance of analysis. We calculated that using a conservative intraclass correlation coefficient of 0·01,^21,22^ and assuming 120 students per school, a trial involving 32 schools per group would provide 80% power to detect a difference of 1·4% at 12–14 months, with a 5% significance level. Informed by our pilot trial,^22^ with 7% attrition (plus an additional two schools to be conservative), our aim was to recruit a total of 66 schools to the trial (n=33 per group), roughly comprising 7900 students.

The analysis was on an intention-to-treat basis and used multilevel, mixed effects logistic regression models for binary outcomes and mixed effects linear regression models for continuous outcomes. All models included cluster (school) as a random effect and used robust SEs, adjusting for the corresponding baseline outcome and stratification variables (FSM and nation). To examine the potential effect of missing data, sensitivity analyses including imputed follow-up data based on the worst performing school (in relation to detected incidence of unprotected sex) and best performing school (where students did not have unprotected sex) at baseline were done for schools where we did not have follow-up data (see appendix p 10). Analysis was done by means of Stata–SE, version 15.1. Significance was defined using a two-sided test with α=0.05.

We assessed differential effects of the intervention on the primary outcome according to subgroups by fitting interaction terms (treatment group by subgroup) in multilevel, mixed effects logistic regression models with p values from a global test for interaction. We did five prespecified subgroup analyses: by student sex; by family-level socioeconomic deprivation, which was measured by means of the Family Affluence Scale; by ethnicity; by nation; and by those who reported having unprotected sex at baseline. An additional post-hoc exploratory subgroup analysis was done on those who were sexually active at baseline. A conservative 99% CI was used in all subgroup analyses.

Three exploratory post-hoc analyses were done after reviewing the primary outcome analyses. These analyses were done to separately analyse effects on the component questions of the primary outcome of avoidance of unprotected sex: sexual abstinence (Have you ever had sex with another person?) and use of reliable contraception at last sex (Last time you had sex, did you use contraception? Last time you had sex, did you or your partner use withdrawal or natural family planning–rhythm method?; figure 1). We did these post-hoc analyses because the sample size calculation for the primary outcome of avoidance of unprotected sex was based on the whole population and an acknowledgement that there are two primary ways of avoiding unprotected sex, namely sexual abstinence or use of reliable contraception once sexually active. The primary outcome combined both of these outcomes. In the post-hoc analyses we looked at these two components separately to allow for the examination of whether the intervention caused a decrease in sexual abstinence (or more pupils becoming sexually active), or promoted the use of reliable contraception for those who were sexually active. The UK-wide population-level data on which the sample size was based informed our assumption that at least a quarter of the sample might be sexually active by age 15 and our assumption for this to continue to incrementally increase during adolescence.^21^ It was thus especially important to also understand whether the intervention was effective among those who were sexually active. Finally, we also examined sex differences (male versus female) in use of reliable contraception.

For the process evaluation, we used thematic analysis for the qualitative data (appendix p 8). For the within-trial economic analysis, the delivery cost of the intervention was calculated from time spent preparing and delivering the intervention multiplied by the average hourly wage of a schoolteacher. This was divided by the number of students in each school randomly assigned to the intervention to calculate the total opportunity cost per student (appendix p 18). A similar method was used to calculate the delivery costs of standard RSE in control schools. Self-reported health resource use for each student was multiplied by unit costs from published sources (appendix p 14). Both the unadjusted and adjusted mean cost per student at follow-up were reported for each group; the adjusted mean cost was obtained after adjusting for baseline resource use and stratification variables, which used clustering (school) as a random effect. The associated 95% CIs were calculated on the basis of bootstrapped bias-corrected methods.

A decision analytic model with a 20-year time horizon estimated the expected costs and consequences for a hypothetical cohort of students with similar characteristics to those who participated in the trial. For the decision model, we prespecified the use of students’ reports of contraception use and reports of STI diagnoses in relation to modelling the number of pregnancies and STIs averted as a result of participation in the trial (see appendix pp 14–18). We calculated the mean incremental total cost of the intervention compared with standard RSE practice for cost per pregnancy averted; cost per STI averted; and cost per quality adjusted life year (QALY) gained over a 20-year time horizon from a health-care (excluding government-funded benefits—ie, child benefits, child tax credits, income support, and housing benefits) and public sector perspective (including government-funded benefits). All benefits and costs after 12 months were discounted at an annual rate of 3·5% to capture time preferences for costs and benefits. Both deterministic and probabilistic sensitivity analyses were done to assess robustness to alternative modelling assumptions (appendix pp 15–23). The trial is registered with ISRCTN registry (99459996).

### Role of the funding source

The funder of the study had no role in study design, data collection, data analysis, data interpretation, or writing of the report.

## Results

**Figure 2:**
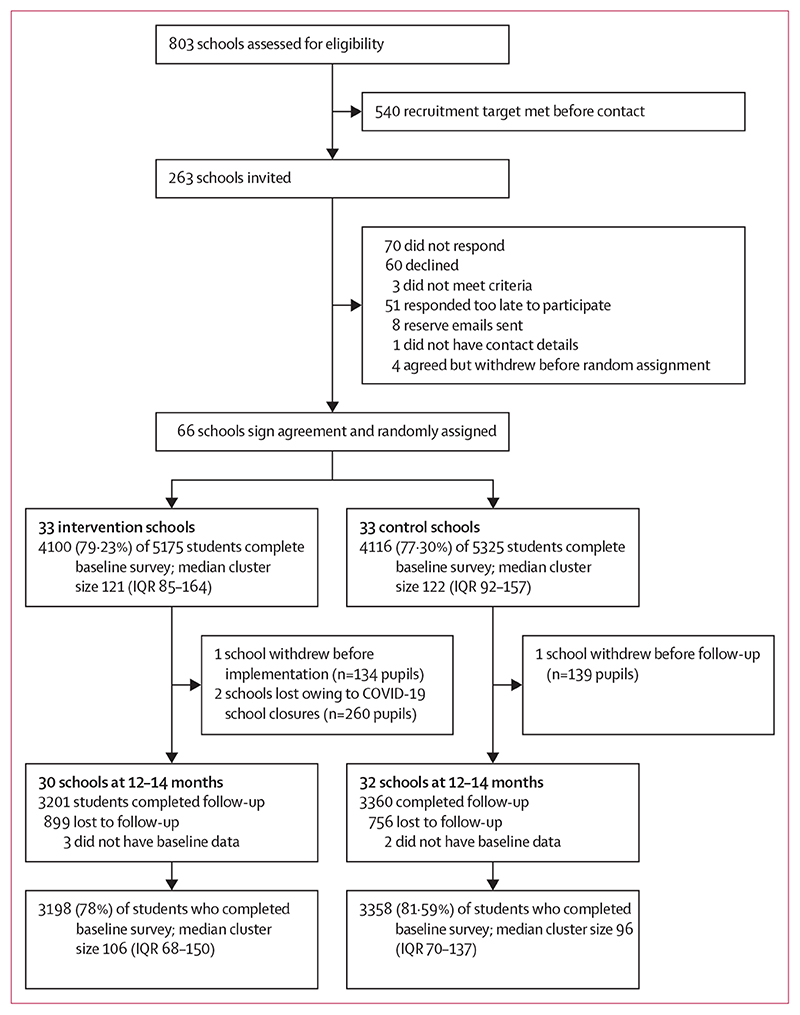
Trial profile

Enrolment took place between Feb 1, 2018 and Sept 1, 2018. The trial was done between Feb 1, 2018, and March 6, 2019. Figure 2 shows the trial profile. Of 803 schools assessed for eligibility, 263 schools were invited to enrol by letter (172, which were above the national FSM entitlement median, and 91, which were below). Positive responses were received from 70 schools. Of these, 66 agreed to be randomly assigned (38 from the above-median FSM stratum and 28 from the below-median stratum). The inclusion of slightly more schools from the above-median FSM stratum reflects the greater need for efforts to reduce unintended pregnancies in areas of higher social deprivation.^24^ 8216 (78·24%) of 10 500 eligible students in participating schools provided data at baseline (4100 [79·23%] of 5175 in the intervention group *vs* 4116 [77·29%] of 5325 in the control group). Table 1 shows that student and school characteristics were similar across groups at baseline. 6556 (79·79%) of those who completed baseline provided data at 12–14 months follow-up (3198 [78·00%] in the intervention group *vs* 3358 [81·59%] in the control group). There was a loss to follow-up of four clusters (one from the control group and three from the intervention group) two of which were owing to school closures as a result of the COVID-19 pandemic (see appendix p 1 for a detailed discussion of losses to follow-up). In this trial, almost 22% of the trial population (mean age 15.5, SD 0·4) had become sexually active, which was somewhat lower than that reported in UK population data for the four nations at age 15 years (25-33%). Furthermore, 2.2% (n=145) of the trial population reported not using reliable contraception at last sex, which is somewhat lower than reported in UK population data (2.8%) for those aged 15 years.

At 12–14 months follow-up, the number of students who reported that they avoided unprotected sex (ie, by means of sexual abstinence or use of reliable contraception at last sex) was 2648 (86.62%) of 3057 in the intervention group and 2768 (86.42%) of 3203 in the control group (adjusted odds ratio [aOR] 0.85, 95% CI 0.58-1.26, p=0.42), indicating no significant effect on the primary outcome (table 2). Exploratory post-hoc analysis of the primary outcome component questions (table 3) showed no effect on self-reported sexual abstinence at 12-14 months (2407 [78.30%] of 3074 in the intervention group and 2511 [78.25%] of 3209 in the control group; aOR 0.85 [5% CI 0.58-1.24], p=0.39). There was, however, evidence that students in the intervention group were more likely than those in the control group to report use of reliable contraception at last sex (42 [39.62%] of 106 in the intervention group *vs*29 [26.36%] of 110 in the control group; aOR 0.52 [95% CI 0.29-0.92], p=0.025). Exploratory post-hoc subgroup analyses on this outcome showed no differences according to participant sex (p=0.34; table 3).

Table 2 shows that for the secondary outcomes, there was evidence that students in intervention schools had greater knowledge about safe methods of contraception and accessing contraception, improved attitudes towards progressive male gender roles, and had stronger intentions to avoid an unintended pregnancy compared with students in control schools. There was no significant difference in sexual self-efficacy or comfort communicating about avoiding unintended pregnancy.

Table 4 shows the subgroup analyses of the primary outcome. The analyses showed no significant difference in the primary outcome for nation, sex, ethnicity, or family-level socioeconomic deprivation.

Sensitivity analysis to examine the potential effect of missing data on the primary outcome was done, but this did not lead to any substantive or significant differences in the results reported (appendix p 10).

The process evaluation assessed trial context, implementation fidelity, and mechanisms of effect. In relation to trial context, the primary reasons for participation from the schools’ perspectives were the perception of the If I Were Jack intervention as a high quality, novel resource, along with a recognised need by schools to address RSE, the recognised gap in relation to engaging males, and the opportunity for teacher training. Conflicting commitments, research fatigue, and the additional time commitment required by the trial were the primary reasons for non-participation. RSE provision, outside of If I Were Jack, in both the intervention and control groups, was broadly similar or equal in terms of the distribution of schools categorised as having high (n=3 [9·38%] *vs* n=3 [9·38%]), medium (n=7 [21·88%] *vs* n=8 [25·00%]), or low (n=19 [59·38%] *vs* n=15 [46.88%]) provision of RSE (appendix p 11). Overall, the control schools were not considered to be contaminated by changes to provision as a result of participating in the trial. Intervention implementation fidelity was generally medium to high across intervention schools (appendix p 12). In relation to perceived mechanisms of effect, students and teachers thought the intervention was an opportunity to gain knowledge on sex and relationships and to acquire skills in relation to sourcing information and support. Teachers and students noted increased confidence among students to communicate with peers about sex and relationships. The gender focus of the intervention, the engagement of males, and the generation of empathy and understanding of both male and female perspectives and challenging of unequal gender norms were regarded as a key strength by both students and teachers. Potential limitations relating to this approach were a perception that the programme could overshadow female perspectives, the heterosexual focus on unintended pregnancy, and an acknowledgment by teachers that a short programme might be insufficient to challenge deeply embedded gender norms and gender inequalities around sexuality.

The within-trial health economic analysis showed that the mean total cost to the education sector of delivering the intervention was *£*5.42 per student compared with *£*4.42 for standard RSE in control schools. Taking account of health-care costs, the total mean incremental cost of the intervention compared with standard RSE was an additional *£*2·83 (95% CI-*£*2·64 to *£*8·29) per student (see appendix pp 18-20). The decision modelling indicated that owing to the greater use of reliable contraception at last sex in intervention than control schools, the If I Were Jack intervention would result in 379 (95% CI 231-477) fewer unintended pregnancies, 680 fewer STIs (95% CI 189 to 1647), and 10 QALYs (95% CI 5 to 16) gained per 100 000 young people over a 20-year time horizon for a cost saving of *£*9.89 (*£*4.83 to *£*15.60) per young person that receives the If I Were Jack intervention compared with standard RSE (Table 5).

There were no serious or adverse events reported in the trial.

## Discussion

The findings from the JACK trial show that a school-based gender-transformative RSE intervention did not have a significant effect on self-reported avoidance of unprotected sex, measured as either sexual abstinence or use of reliable contraceptive at last sex. However, in post-hoc exploratory analysis of these questions separately, the findings show significant positive differences between the intervention group compared with the control group of self-reported use of reliable contraception at last sex for those who already were, or became sexually active by the time of follow-up. There were no significant differences in rates of self-reported sexual abstinence between the intervention or control groups at the 12–14 month follow-up. The intervention led to significant improvements in outcomes linked to our theory of change: sexual health knowledge, progressive male gender norms, and intentions to avoid an unintended pregnancy. We did not find evidence of significant improvements in sexual self-efficacy or comfort communicating about avoiding unintended pregnancy. The intervention was relatively cheap, falling into the very low-cost category for UK school interventions^25^ at an additional cost of *£*2.83 per student, and over a 20-year period is likely to lead to a cost saving of *£*9.89 per adolescent receiving the intervention compared with existing RSE.

The potential effectiveness of If I Were Jack in increasing self-reported use of reliable contraceptive among students who were sexually active at baseline or by follow-up could be important at the population-level given the scalable nature of school-based interventions and the incremental increase in sexual initiation during adolescence. Previous high quality systematic reviews of randomised trials^6,9,11,26^ report few school-based RSE interventions that are effective in increasing adolescent contraceptive use at last sex, and none of the previous UK-based trials of RSE interventions showed effectiveness on this outcome.^27^ The results of the present study indicate that the intervention might be effective for groups at higher risk of unintended pregnancy owing to earlier sexual initiation,^28^ and highlight the relevance of the intervention in promoting contraceptive use among students who already were or became sexually active by age 15 years. Additionally, the results indicate that the If I were Jack intervention, which is based on a comprehensive approach to RSE, did not lead to increases in adolescent sexual initiation.

The US-based CAS-Carrera^29^ randomised trial found that their RSE intervention was effective in increasing contraceptive use among adolescent females. We found that If I Were Jack was equally effective in promoting the use of reliable contraceptives at last sex for males and females, which is suggestive of the importance of male engagement gender-transformative components.^8^ Moreover, as predicted by our theory of change, we found that the intervention improved young people’s intentions to avoid unintended pregnancy, which includes improvements in their perceived ability to share responsibility for contraceptive decision making with a partner, and to communicate consent for sexual intercourse, sexual readiness, and sexual preferences regarding timing. These are important effects because they address a neglected research area of considering young people’s sexual desires and preferences as part of healthy relationships in RSE.^11^ Although RSE specialists have called for the inclusion of a discourse of pleasure to enhance a more holistic view of sexual wellbeing and other aspects of positive sexuality,^11,18^ the measurement of these outcomes is “conspicuous by their absence”^11^ in previous randomised trials of RSE, especially in relation to pregnancy prevention.^30^

We found that If I Were Jack, a school-based, teacher-delivered intervention, could be cost-effective over the long term, even with modest increases in contraceptive use among sexually active adolescents, with no effects on rates of sexual initiation. These results are consistent with the findings of a pioneering health economic evaluation of school-based RSE in the USA^31^ and support the latest UNESCO report^13^ on the cost of delivering school-based RSE. Our inclusion of a health economic analysis addresses an international deficit in health economic evaluations of RSE, and specifically school-based RSE, identified in the Guttmacher-*Lancet* Commission on sexual and reproductive health and elsewhere.^2–4,18,32^ However, the results should be interpreted with caution given the range of assumptions modelled to project the results to 20 years.

In terms of strengths, our research team was independent from the intervention delivery team, random allocations were only revealed to schools after baseline data had been collected, and fieldworkers remained masked to the allocation throughout the trial. All outcomes were assessed by means of age-appropriate, and validated instruments (where available). In order to improve consistency across students in reporting, we provided definitions for sexual activities, and student questionnaires were administered under examination conditions to enhance confidentiality.^12^ The JACK trial was done in a group of schools that are ethnically, culturally, and socioeconomically diverse. Uniquely, in a trial of an RSE intervention, we included schools in the four nations of the UK and schools that are faith based (though all Christian) as well as those that are not. It is also one of the few trials which assessed gender norms and presents disaggregated results by participant sex. ^12^

In terms of limitations, although school dropout and student absences could have introduced bias, attrition rates were similar between groups, resulting in an unclear influence on effectiveness but a probable loss of precision in the effect estimates. The observed intraclass correlation coefficient in the current study was much larger than that used in our sample size calculation, despite being based on our own pilot trial^22^ and previous research in this area,^10^ which might have led to some of our analyses being underpowered. It is not feasible to validate young people’s self-reporting of sexual activity with an objective measure. To address limitations, we provided definitions for sexual activities and terminology to aid comprehension, and student questionnaires were administered by fieldworkers under examination conditions to enhance confidentiality of responses. Although the trial included schools with diverse socioeconomic characteristics, it was done in a high-income (UK) setting and is not necessarily generalisable beyond this context. We have studies underway to develop and test the feasibility of adaptations of the If I Were Jack intervention in South America and southern Africa, taking learning from the current trial into account.

In conclusion, we did not find a significant effect on the primary outcome of a reduction in unprotected sex, measured as a combination of sexual abstinence or use of reliable contraception at last sex. We did find positive effects for If I Were Jack in terms of important secondary outcomes of increased sexual health knowledge, improved attitudes, and intentions to support healthy, positive, gender equitable intimate relationships, as well as an increase in use of reliable contraception among adolescents who were, or became, sexually active by follow-up, 12-14 months after the intervention. If I Were Jack is a single, brief, scalable intervention of relatively low cost. That the intervention was found to be potentially cost-effective over the long term, owing to increases in reliable contraceptive use among adolescents as they became sexually active, is an important piece of evidence for public health policy. The added value to the advancement of RSE practice arising from this trial is in showing increased reliable use of contraception, sexual health knowledge, and gender equitable attitudes for males and females through male engagement and gender-transformative programming, which could also be integrated into wider RSE. School-based RSE interventions, such as If I Were Jack, could be one of the most efficient ways of reducing unintended pregnancies and sexually transmitted infections in adolescence, because of their potential to promote contraceptive use in a population-wide, replicable, and sustainable fashion.

## Supplementary Material

Appendices

## Figures and Tables

**Table 1:** Baseline characteristics

	Intervention (n=4100 students; 33 schools)	Control (n=4116 students; 33 schools)
**School characteristics**		
Nation		
England	7 (21.21%)	7 (21.21%)
Northern Ireland	12 (36.36%)	l2 (36.36%)
Scotland	7 (21.21%)	7 (21.21%)
Wales	7 (21.21%)	7 (21.21%)
School sex mix		
Mixed	27 (81.82%)	32 (96.97%)
Boys only	2 (6.06%)	1 (3.03%)
Girls only	4 (l2.l2%)	0
Faith-based schools		
Faith-based	7 (21.21%)	8 (24.24%)
Non-faith-based	26 (78.79%)	25 (75.76%)
Range of students entitled to FSM, %	2-52%	3–44%
**Student characteristics**		
Age, years	14.5 (0.4)	14.5 (0.4)
Sex		
Male	1980 (48.29%)	2121 (51.53%)
Female	2120 (51.70%)	1994 (48.45%)
Ever had sex with another person		
Yes	234 (5.85%)	251 (6.28%)
No	3767 (94.15%)	3743 (93.72%)
Sexual orientation		
Heterosexual	3652 (90.08%)	3722 (91.45%)
Homosexual	45 (1.11%)	44 (1.08%)
Bisexual	174 (4.29%)	131 (3.22)
Unsure	85 (2.10%)	73 (1.79%)
Prefer not to say	59 (1.46%)	65 (1.60%)
Other	39 (0.96%)	35 (0.86%)
Race		
White	3114 (76.52%)	30.79 (75.47%)
Asian, Asian British–Irish	475 (11.67%)	326 (7.99%)
Black African, Black Caribbean, Black British–Irish	255 (6.26%)	369 (9.04%)
Mixed–Multiple ethnic backgrounds	l55 (3.81%)	l80 (4.41%)
Other	71 (1.74%)	126 (3.09%)
Religion		
No religion	1643 (40.96%)	1327 (33.03%)
Roman catholic	743 (l8.52%)	1074 (26.73%)
Protestant	997 (24.86%)	1089 (27.10%)
Buddhist	11 (0.27%)	20 (0.50%)
Jewish	13 (0.32%)	6 (0.15%)
Muslim	417 (10.40%)	362 (9.01%)
Sikh	25 (0.62%)	32 (0.80%)
Other	162 (4.04%)	108 (2.69%)
Religiosity		
Very religious	260 (6.38%)	324 (7.95%)
Fairly religious	1053 (25.85%)	1175 (28.81%)
Not very religious	1043 (25.61%)	1140 (27.95%)
Not at all religious	1717 (42.16%)	1439 (35.29%)
**Socioeconomic status**		
Cluster level		
Schools above FSM median	20 (60.61%)	19 (57.58%)
Schools be10w FSM median	13 (39.39%)	14 (42.42%)
Individual level		
Family Affluence Scale	6.1 (109)	6.1 (109)
low tertile	1495 (36.69%)	1445 (35.34%)
Medium tertile	1617 (39.68)	1656 (40.50%)
High tertile	963 (23.63%)	988 (24.16%)
**Educational aspirations**		
Expected age leaving school		
16 years	756 (18.54%)	7.2 (17.16%)
18 years	2372 (58.18%)	2474 (60.49%)
I don’t know yet	949 (23.38 %)	914 (22.35%)
Aspiration on leaving school		
Getting or trying to get a job	811 (20.38%)	829 (20.69%)
Be in a job training scheme or apprenticeship	330 (8.29)	383 (9.56%)
Be at university	1893 (47.56%)	2.22 (50.46%)
Be at a further education college (studying for a trade–job)	784 (19.70)	623 (15.55%)
Be a full-time mum or a dad	15 (0.38%)	11 (0.27%)
Other	147 (3.69%)	139 (3.47%)

Data are n (%) or mean (SD). FSM=free school meals. Percentages are based on the number of respondents who answered each question.

**Table 2:** Primary and secondary outcomes at 12–14 months

	N	Intervention group	N	Control group	Adjusted odds ratio (95% CI)* or mean difference (SD)	p value	Intraclass correlation coefficient
**Primary outcome**							
Avoidance of unprotected sex (sexual abstinence or use of reliable contraception at last sex)	3057	2648 (86.62%)	3203	2768 (86.42%)	0.85 (0.58 to 1.26)	0.42	0.118
**Secondary outcomes** †							
Knowledge score	3198	1.09 (1.92)	3358	0.87 (1.90)	0.18 (0.024 to 0.34)	0.02	0.030
Male roles attitudes score	2999	-0.35 (4.16)	3146	-0.26 (4.07)	-0.33 (-0.64 to -0.02)	0.04	0.019
Comfort communicating score	3053	0.50 (1.92)	3186	0.37 (1.98)	0.00 (-0.11 to 0.12)	0.95	0.009
Sexual self-efficacy score	3063	0.13 (0.45)	3202	0.09 (0.48)	0.02 (-0.003 to 0.05)	0.08	0.004
Intentions to avoid a teenage pregnancy score	3075	2.49 (8.80)	3228	1.72 (8.75)	0.61 (0.16 to 1.07)	0.01	0.008

Data are n (%), mean (SD), or adjusted odds ratio.

* Adjusted for corresponding outcome at baseline, nation and above, or below median percentage of students eligible for free school meals.

† Mean change from baseline (SD).

**Table 3:** Exploratory post-hoc analyses of primary outcome components

	N	Intervention group	N	Control group	Adjusted* odds ratio (99% CI)	p value	Intraclass correlation coefficient
Sexual abstinence	3074	2407 (78.30%)	3209	2511 (78.25%)	0.85 (0.58-1.24)	0.39	0.122
Use of reliable	106	42 (39.62%)	110	29 (26.36%)	0.52 (0.29-0.92)	0.025	0.000
contraception at last sex							
Male	46	35 (76.09%)	69	52 (75.36%)	0.82 (0.23-2.92)	0.34†	0.000
Female	60	29 (48.33%)	41	29 (70.73%)	0.4l (0.13-1.34)	··	··

Data are n (%). Percentages are based on numbers within each subgroup who reported the outcome.

* Adjusted for primary outcome at baseline, country and above, or below median percentage of students eligible for free school meals.

† Global test for interaction.

**Table 4:** Subgroup analyses for the primary outcome at 12–14 months

	N	Intervention group	N	Control group	Adjusted odds ratio (99% CI)	Interaction p value*	Intraclass correlation coefficient
Sex†							
Male	1475	1264 (85.69%)	1658	1421 (85.71%)	0.84 (0o49-1.42)	0.81	0.119
Female	1582	1384 (87.48%)	1545	1347 (87.18%)	0.87 (0.49-1.56)	··	··
Family Affluence Scale‡	··	··	··	··	··	0.60	0.117
Low tertile	1113	976 (87.69%)	1081	947 (87.60%)	0.83 (0.46-1.47)	··	··
Medium tertile	1193	1050 (88.01%)	1298	1127 (86.83%)	0.80 (0.46-1.41)	··	··
High tertile	742	614 (82.75%)	813	683 (84.01%)	0.97 (0.52-1.81)	··	··
Ethnicity†	··	··	··	··	··	0.16	0.058
White§	2351	1973 (83.92%)	2370	1981 (83.59%)	0.86 (0.58-1.27)	··	··
Asian	369	361 (97.83%)	279	274 (98.21%)	1.43 (0.31-6.55)	··	··
Black	169	157 (92.90%)	302	288 (95.36%)	1.69 (0.55-5.21)	··	··
Other¶	149	141 (94.63%)	238	212 (89.08%)	0.43 (0.11-1.65)	··	··
Nation∥	··	··	··	··	··	0.05	0.054
Northern Ireland	689	581 (84.33%)	976	811 (83.09%)	0.78 (0.41-1.51)	··	··
Scotland	626	533 (85.14%)	527	426 (80.83%)	0.76 (0.45-1.28)	··	··
England	734	707 (96.32%)	942	861 (91.40%)	0.46 (0.18-1.13)	··	··
Wales	1008	827 (82.04%)	758	670 (88.39%)	1.68 (0.72-3.91)	··	··
Having had unprotected sex at baseline**	··	··	··	··	··	0.27	0.119
Yes	107	42 (39.25%)	124	42 (33.87%)	0.64 (0.28-1.46)	··	··
No	2950	2606 (88.34%)	3079	2726 (88.54%)	0.87 (0.52-1.47)	··	··
Sexually Active at baseline††	··	··	··	··	··	0.06	0.116
Yes	154	75 (48.70%)	171	68 (39.77%)	0.56 (0.29-1.09)	··	··
No	2903	2573 (88.63%)	3032	2700 (89.05%)	0.89 (0.53-1.51)	··	··

Data are n (%) unless stated otherwise. Percentages are based on numbers within each subgroup who reported the primary outcome.

* Interaction p value is from a global test for interaction.

† Adjusted for primary outcome at baseline, country and above or below FSM median.

‡ Adjusted for primary outcome at baseline and country.

§ Includes White English, Irish, Northern Irish, Scottish, Welsh, British, and any other White background.

¶ Includes mixed or multiple ethnic background and other.

∥ Adjusted for primary outcome at baseline and above or below FSM median.

** Adjusted for country and above or below free school meal median.

†† Exploratory post-hoc sub-group analysis.

**Table 5:** Estimated number of averted unintended pregnancies, STIs, and QALYs loss, and incremental costs per 100 000 students over a 20-year time horizon

	Intervention group	Control group	Difference
Unintended pregnancies	2152	2531	379
STIs	1173	1853	680
QALYs loss	18	28	10
Incremental health and social care costs			
Total costs (without state benefits)	£19 470 336	£20 459 742	-£989406
Total costs (with state benefits)	£166111565	£191 457 170	-£25 345 605

STI=sexually transmitted infection. QALY=quality adjusted life year.

## Data Availability

Queen’s University Belfast, the sponsoring organisation, is the custodian of all data collected during the study. The Principal Investigator (PI), Professor Maria Lohan, controls the use, publication and copyright of the project data. All data will be publicly archived in the UK Data Archive located at the University of Essex in 2026. In the interim, all data requests should be submitted to the corresponding author for consideration. Access to anonymised data (with data dictionary) might be granted following review by the principal investigator and Trial Management Group.

## Research in context

### Evidence before this study

Systematic reviews have shown that school-based relationships and sexuality education (RSE) can be effective in equipping adolescents with the knowledge and skills for avoiding unintended pregnancies, but might not reduce unprotected sex. Comprehensive RSE is shown to be more effective than so-called abstinence-only approaches. A notable gap identified in reviews of RSE education is the use of gender-transformative components that seek to challenge the gender inequalities that affect sexual and reproductive health and rights (SRHR) alongside intentional male engagement to promote male and female joint responsibility for sexual and reproductive health. In 2018, we did an evidence and gap map and systematic review of reviews of experimental or quasi-experimental intervention studies engaging males in relation to all WHO-defined SRHR outcomes. We searched CINAHL, Medline, PsycINFO, Social Science Citation Index-expanded, Cochrane Library, Campbell Collaboration, Embase, Global Health Library, Scopus, and Google Scholar (Jan 1 2007-July 31, 2018 with no language restrictions) using a prepublished search strategy incorporating MeSH terms for “SRHR” and “Males/Masculinities”. We included 462 reviews, and identified that only 39 (8·4%) included gender-transformative interventions. In 2019, we did another systematic review of gender-transformative intervention studies engaging males to improve SRHR, on the basis of this subset of 39 reviews. We identified no randomised trials of male-engagement gender-transformative school-based RSE to reduce adolescent pregnancy. A further notable gap identified in systematic reviews is in relation to cost-benefit analyses of RSE.

### Added value of this study

The JACK trial is the first cluster-randomised trial with embedded process and health economic evaluations of a school-based, gender-transformative, RSE intervention. The intervention If I Were Jack aims to promote joint responsibility in boys and girls aged 14-15 years in preventing unintended pregnancies and increasing positive sexual health. We found the intervention had no significant effect on the primary outcome of reducing unprotected sex among all students measured as sexual abstinence or use of reliable contraception at last sex. However, in post-hoc exploratory analysis of the two separate components of the primary outcome, we found significant increases in the use of reliable contraception at last sex among students who were or became sexually active by 12-14 months after the intervention. There was no significant difference between the groups for sexual abstinence. The intervention had a positive effect on all students’ sexual health knowledge, support for progressive male role attitudes, and intentions to prevent unintended pregnancy. There was, however, no effect on communication or sexual self-efficacy skills. If I Were Jack is low cost compared with other educational interventions and modelling based on the observed increases in reliable contraceptive use suggests that the intervention is likely to be cost-effective over a 20-year period through the avoidance of unintended pregnancies and sexually transmitted infections.

### Implications of all the available evidence

Comprehensive school-based RSE interventions can have effects of public health importance in relation to improving adolescents’ foundational knowledge for safe, positive, and pleasurable intimate relationships now and in the future. In particular, there is great interest in the potential of gender-transformative sexual education. This trial did not find evidence that such an intervention reduces the likelihood of unprotected sex in 14-16 year olds by increasing sexual abstinence or reliable use of contraception. However, the inclusion of intentional male-engagement and gender-transformative programming that challenge gender inequalities was found to increase the use of reliable contraception among students who were sexually active at a young age, and as a result is likely to be cost-effective. Future studies should consider the further development and evaluation of male engagement and gender-transformative components in RSE curricula at other ages and explore the longer-term effects of these interventions as more students become sexually active.
