# Supplementary material for: Effects of gender-transformative relationships and sexuality education to reduce adolescent pregnancy (the JACK trial): a cluster-randomised trial
Source: Lancet Public Health. Author manuscript; Available in PMC 2022 Dec 20. (PMC7613971; doi:10.1016/S2468-2667(22)00117-7)
Supplement: Appendices [file EMS158437-supplement-Appendices.pdf]

THE LANCET
Public Health

Supplementary appendix

This appendix formed part of the original submission and has been peer reviewed. We post it as supplied by the authors.

Supplement to: Lohan M, Brennan-Wilson A, Hunter R, et al. Effects of gender-transformative relationships and sexuality education to reduce adolescent pregnancy (the JACK trial): a cluster-randomised trial. *Lancet Public Health* 2022; **7:** e626–37.

Appendix 1

1. Losses to follow-up

School-level attrition from trial

Retention at school level was generally strong. However, four recruited schools (533 pupils; 6.5% of pupils enrolled in the trial) who completed baseline surveys were lost to follow-up (three in the intervention and one in the control arm).

- The first school withdrew prior to implementation (intervention arm, England, 134 pupils (1.6%), all girls, in above median FSM stratum (34% of pupils in receipt of FSM) and non-faith-based). Withdrawal reasons were teachers' reluctance to engage with teacher training and additional commitments associated with being 'a case-study school', involving observations and interviews.
- The second school withdrew after implementation of intervention but before follow-up surveys were conducted (control arm, Wales, 139 pupils (1.7%), co-educational, in above median FSM stratum (28.6% pupils in receipt of FSM), non-faith-based). Withdrawal reasons cited were 'due to other commitments with year 11 at this time'.
- The final two schools withdrew owing to the Covid-19 pandemic lockdown measures. The UK lockdown (from March 2020) led to school closures, making it impossible to visit and collect survey data from two schools. Both schools were in the intervention arm and had implemented the programme. One school was in NI (164 pupils (2.0%), co-ed, in above median FSM stratum (20% of pupils in receipt of FSM), faith-based); the other school was in Scotland (96 pupils (1.2%), co-ed, in the below median FSM stratum (6% of pupils in receipt of FSM; non-faith-based).

Pupil-level attrition from trial

441 pupils (4.2% of eligible pupils) were opted out of participation in the research by parents before baseline data collection. A total of 8216 pupils (n=4100 intervention; 4116 control) completed baseline and 6561 (n=3201 intervention; n=3360 control) completed follow-up. Therefore, 1655 pupils (20.1% of pupils enrolled in the trial) were lost to follow up at this stage. The primary reason accounting for the 20.1% loss to follow-up from baseline to follow-up was pupil absence or pupils having left the school (n= 1088; 65.7% of losses to follow-up). The remaining losses to follow-up were due to school withdrawal (n= 273; 16.5%), school closures due to the Covid-19 pandemic (n= 260; 15.7%), and pupil opt-out (n= 34; 2.1%).

Risk of bias

Losses to follow-up were assessed to determine any potential risk of bias. Specifically, we queried whether there were more losses to follow-up in certain types of schools based on location, eligibility for free school meals and whether or not the school was faith-based. We also assessed whether there were differences in losses to follow-up between the two trial arms.

The rate of loss to follow-up was lowest in Wales (15.8%) and similar across the other three nations (approx. 22%). There was very little difference in loss to follow-up in relation to the proportion of pupils eligible for FSM in schools (below the nation-level median = 20.3%; above 20.1%) and loss to follow-up for faith-based and non-faith-based schools was almost identical (approx. 37%). There was only a small difference in relation to parent opt-out (at the point of recruitment) for faith-based (13.2%) and non-faith-based schools (10.7%). Differences in losses to follow-up for the intervention (n=899, 21.9%) and control groups (n= 756; 18.4%) were also small.

2. Deviations to trial protocol and rationale

The protocol for this evaluation was published in June 2018. A small number of minor deviations to the original Protocol published in June 2018 have been made and these are detailed below.

Minor changes to recruitment timing

The published protocol stated that the school recruitment period would run from February to June 2018. However, in order to recruit the full number of schools, this was extended slightly, and school recruitment took place over a six-month period (February–June and to end September 2018), with a break during the summer period (July and August).

Minor changes to intervention implementation

Our published protocol stated that:

As part of intervention implementation, there will be a standardised 60 min training session for RSE teachers implementing the intervention. The training session will adhere to a predefined teacher-trainer protocol and will be delivered in schools by nation-specific established statutory and non-statutory RSE co-ordinators who normally provide RSE teacher training in schools.

We encountered difficulties in using RSE coordinators who normally provide RSE in schools in Scotland and England. In Scotland, Education Scotland was unable to provide us with this teacher trainer resource. In England, the non-statutory facilitator did not complete training in two of the six schools in the intervention arm. To address both these issues, the project team appointed external teacher trainers. These teacher trainers were trained in the same way as all other teacher trainers as per protocol. The fidelity of the teacher training offered by the replacement teacher trainers was also assessed along with all other teacher trainers as part of trial methodology.

Minor changes to data collection

There was a change in timing in the collection of Teacher Implementation Logs and Teacher Resource Use Questionnaires. These data were planned for collection following implementation. However, teacher time and workload made it difficult to accommodate this. As a compromise, and to avoid the risk of retrieving no data, flexibility on timing of receipt was introduced and it was agreed that, at a minimum, a single Implementation Log/Teacher Resource Use Questionnaire would be completed on behalf of a given school.

Additionally, it was not possible to arrange and collect focus group data from parents as specified in the original protocol. Parental engagement limited opportunity to conduct focus groups, thus individual face-to-face and telephone interviews were held in place of these.

Minor additions to data collection

In addition to the published aims, a Student Engagement Questionnaire (SEQ) was also added post-protocol to the process evaluation in all intervention schools. The SEQ was distributed by teachers to pupil participants in the intervention arm in 32 schools (n=3179 pupils) across the four nations of the UK upon completion of the intervention. The SEQ included questions on pupil demographics and implementation factors (delivery length/mode, parental participation, and teaching quality) and included a six-item student engagement questionnaire (which showed good reliability and validity with a Cronbach's alpha = 0.85). It also included three open-ended questions, which asked pupils their opinions on the IVD, as well as their most and least favourite programme activities, and reasons.

Minor changes to analysis plan/analysis principles

Analysis of individual level socio-economic analysis was stated in published protocol as being measured by 'highest qualification level of parents'. However, this was as a drafting error. It was not intended to use this measure for two reasons. First, it was found to be unreliable in the feasibility trial owing to a large amount of missing data. Second, it was predicted to be unreliable due to the non-standardisation of educational terms for highest school qualification (e.g. A-levels and Scottish Highers). This was replaced with the Family Affluence scale (number of family holidays during the past 12 months, family computer(s), ownership of a family car, and ownership of a dishwasher). The data was analysed in terms of a tertile of low, medium and high. Other measures also included were pupils' highest educational aspirations, and age expected to leave school.

3. Jack Trial Logic Model

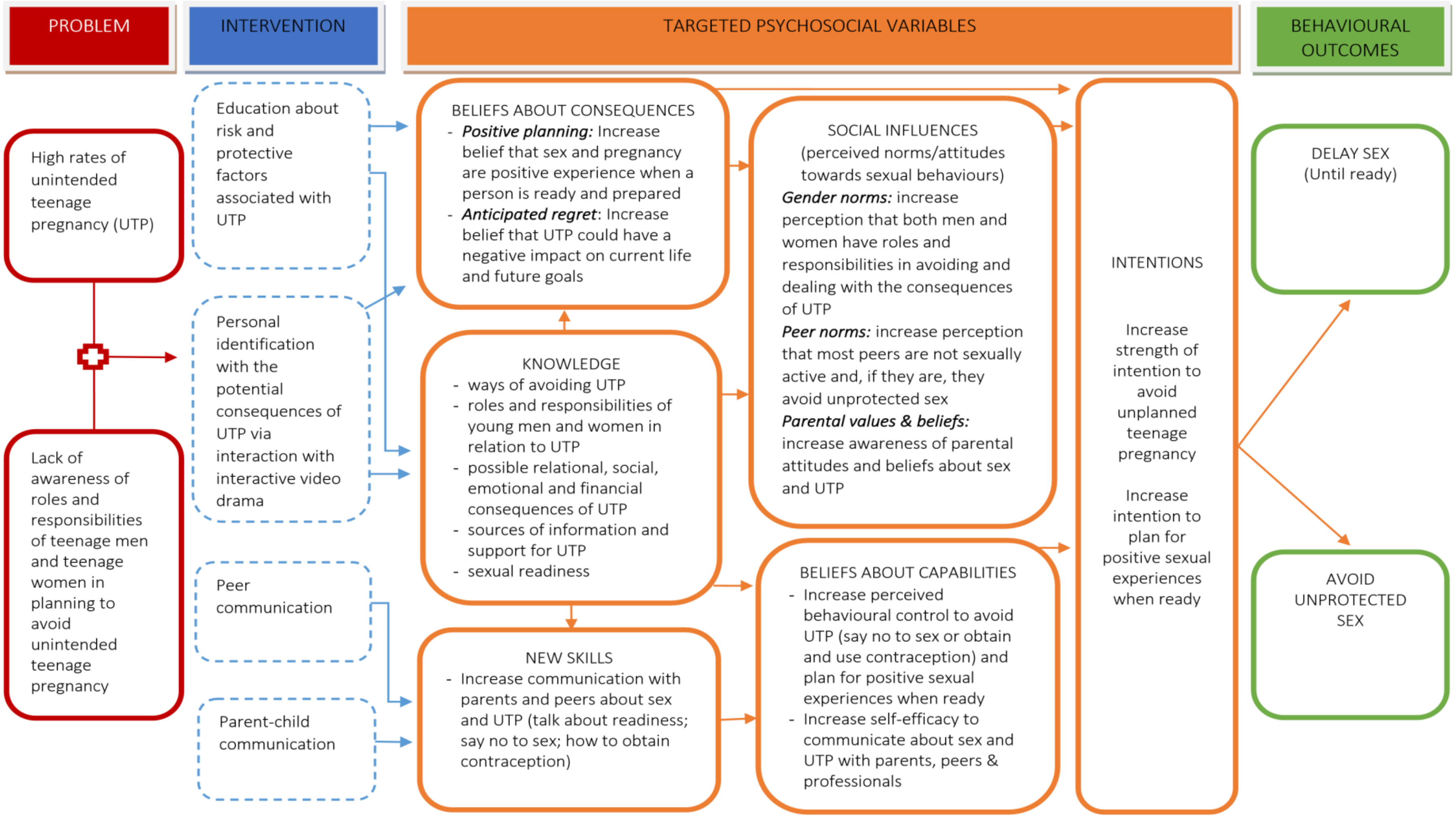

***Figure 1** Theory of Change Logic Model of the* If I Were Jack *intervention*

4. The Template for Intervention Description and Replication (TIDieR[1]) checklist: *If I Were Jack*:

***Name and brief description*:**
If I Were Jack is an evidenced-based, user-informed educational resource designed to prevent unintended pregnancy by increasing adolescents' intentions to avoid unprotected sexual intercourse. It is a gender-transformative intervention especially designed to provoke critical reflection on the gendered norms of teenage pregnancy and to encourage males to share reproductive responsibility while not excluding female perspectives. In development since 2012, with a further optimisation phase in 2017 as part of the Jack Trial, it has involved sustained communication and co-design with target groups, including pupils, teachers and parents, along with a wide range of stakeholders across the four nations of the UK and Ireland. A cluster RCT and transferability to assess its acceptability and feasibility and cost of delivery was published in 2018[2].

Why? Rationale of essential elements:
If I Were Jack is built on an innovative set of programme components which previous research has identified as contributors to effective Relationships and Sexuality Education (RSE). These include:

1. Active-learning activities targeting theoretically informed behavioural and psycho-social correlates of risk behaviour including knowledge, attitudes, perceptions of risk, peer and gender norms, self-efficacy in communication and intention to avoid sexual risk-taking behaviour and to make positive sexual health choices, noted in the logic model as increasing the view that sexual activity and pregnancy can be a positive experiences when ready. Intervention activities are focussed on helping young people to think for themselves on how current or future relationships can be consensual, equitable and pleasurable. For example, through classroom activities, young people are invited to consider what sexual readiness is and how it can be communicated in different relationship scenarios and to offer advice to each other on a fictional student helpline. The planned classroom activities are designed to allow young people to engage in pro-social peer conversations in small groups to obtain information, practice communication scripts and imagine relationship scenarios.
2. Gender-transformative theory and male engagement to address gender inequalities in sexual and reproduction health responsibility: The intervention invites young adolescents to engage in young men's perspectives while equally inviting young adolescents to challenge gender inequalities associated with male sexual desire and female reproductive responsibility. It is designed to promote joint responsibility in preventing an unintended pregnancy and planning for safe and pleasurable sexual relationships when ready for both sexes. A central way of achieving this aim is through addressing masculinities in the opening interactive film. The interactive film depicts the scenario of an unintended pregnancy in a young man's life (described in more detail below). Students engage with questions of masculinities and have to answer questions about how *they* might feel or act. For example, Jack, the lead protagonist, states, "I know it is her body and her choice". However, Jack also acknowledges that he is involved too and acknowledges that he may need support himself to help cope with this difficult situation alongside his girlfriend. Young men and women are invited to consider what they might do if they were in Jack's shoes which vary from responses representing 'restrictive' or patriarchal masculinities to 'gender-equitable masculinities'. Examples of restrictive masculinities are "Do I just want to clear off and pretend it never happened?" and statements he imagines his friends might say, such as "At least you know you are not firing blanks" and "It's her problem, not yours." Examples of more gender-equitable masculinities depicted are when Jack goes along with his girlfriend to a counselling session at her request, and is shown as being supportive of her decision-making; thinking aloud about how she might be feeling; and depictions of Jack engaging alone in working out the pros and cons of pregnancy outcome options and the potential implications for both of them, while acknowledging it is the young woman's choice. Follow-up classroom materials also focus on 'pause, rewind' activities and help students to consider for themselves how to avoid this situation. The classroom activities further encourage communication and behavioural skills among adolescent boys and girls to discuss contraception and consent, to know how to obtain safe contraception and to know how to seek help in the event of an unintended pregnancy. Activities and associated teacher training encourage students to think how intimacy, sexual relationships, and future parenthood are not negative experiences, but something to look forward to and can be pleasurable when one is ready.
3. While a central focus is on engaging boys and provoking discussion about the role of masculinities in gender-equality in sexual and intimate relationships, a careful balance is attempted throughout the programme not to exclude adolescent girls' perspectives. Young people are invited to the challenge of putting themselves in Jack shoes as part of the interactive film, but are also asked to think about what Emma [Jack's girlfriend] might be thinking as the story unfolds. By encouraging adolescent boys and girls to put themselves in Jack shoes, this 'thinking like the other sex' exercise is designed to show how gender ideologies are not necessarily fixed to one sex or the other, but rather that they are relational and

malleable. In the classroom activities, young people get to discuss and probe, in their own terms, male privilege in society as well as how broader social norms such as social class and religious based norms influence sexuality and relationships. The limitation of the heterosexual focus on unintended pregnancy is acknowledged as part of programme delivery by teachers. The broader lessons of safe, consensual and gender-equitable relationships to diverse sexualities is also introduced by teachers throughout the programme, while also not making claims to adequately address all RSE needs.

4. Integration of interactive media by using an age-appropriate and culturally relevant interactive video drama (IVD). Participants are asked to engage in anticipatory thinking on their own and imagine he/she is Jack, a young man who discovers his girlfriend is unintentionally pregnant. Participants are asked to make decisions about what they would do if *I* were Jack, what I would say to my girlfriend, what I think she might be feeling; what my friends might say, what my parents might say, how I might seek help; what do I think about pregnancy outcome options, and how I could help prevent an unintended pregnancy occurring in my life. The IVD was scripted, cast and produced in close consultation with young people and RSE experts. Bespoke versions of the IVD were made, one for Northern Ireland (NI) and Scotland and one for England and Wales to enhance cultural identification with the IVD.
5. Facilitating linkages with sexual and reproductive support services. This is included through role modelling access to services (for males and females) in IVD and upskilling young people in finding local services and provision of information on local services through guided internet searches and distribution to each student of a wallet cards of local adolescent-friendly services.
6. Teacher training in relation to the intervention and teenage pregnancy by trained facilitators in a face to face 90 minute session and the provision of detailed classroom lesson plans. A recorded online version of teacher training is also included.
7. Parental engagement through dedicated online materials co-designed with parents and teachers for parents and sent out by school. In addition, there is and an optional pupil homework assignment for pupils to choose to do with parent/guardian or other carer.

What? A description of the materials:
(i) Ninety-minute face-to-face training session for teachers provided by a trained facilitator.

(II) The *If I Were Jack* opening interactive video drama (IVD) is a culturally sensitive film (locally filmed in both NI and England) intended to immerse teenagers in a story of a week in the life of Jack, a teenager who has just been told his girlfriend is pregnant.

(iii) Classroom materials for teachers containing detailed lesson plans for classroom-based and homework activities. Lesson plans provide pupils with opportunities to source sexual health information and provide a wallet card of local sexual and reproductive health services and are focussed on opportunities for pro-social peer discussion, skills practice, reflection and anticipatory thinking.

(iv) Online materials (brief animation videos) for parents/guardians texted by school to parents to inform them of the intervention and to involve them in the intervention.

(v) Information brochures and factsheets about the intervention and unintended teenage pregnancy for schools, teachers, teacher trainers, young people and parents.

Who? Delivery personnel:
Trained RSE teachers

***How? Modes of delivery*:**
To be delivered during four or six consecutive RSE lessons in classroom settings (depending on normal class durations in differing jurisdictions). The IVD is to be delivered on individual computers/tablets with headphones or projected to the class through a single screen with individual paper worksheets, where technology is limited.

***Where? Locations where intervention has occurred*:**
In second-level schools in Northern Ireland, Scotland, Wales and England. In schools in Ireland, using a further locally produced IVD. A version of the IVD has been delivered in South Australia by the RSE provider Shine. An Irish language version of the classroom materials (with an English language version of IVD) has been piloted in Irish-medium schools. An Irish language version of IVD is now being produced. The intervention has been delivered in faith-based and non-faith-based schools and in mixed sex as well as single sex schools. Translations and cultural adaptations of the intervention are underway in Uruguay and Southern Africa.

5. Description of study instruments: secondary outcomes

The items that make up each of the secondary outcomes scales, as well as the scoring procedure for each scale are described.

Knowledge

Composite measure based on items from the Mathtech Knowledge Inventory SKATA[3,4]

The response options for 'Knowledge' items 1-7 were True/False. The response options for items 8 and 9 were open (numeric or text). Correct responses were coded 1; incorrect responses were coded 0. A total knowledge score was derived by totalling the responses to each question, giving a possible score of 0-9, where higher scores indicate greater knowledge.

1. If a young woman under 16 tells a doctor she may be pregnant, legally the doctor must tell her parents/carers.
2. All methods of contraception protect against sexually transmitted infections (STIs).
3. A woman can get pregnant even if the man withdraws his penis (pulls out) before ejaculation (cuming).
4. Contraception (when used correctly) provides as much protection against pregnancy as not having sex.
5. The withdrawal method (when the man pulls out before cuming) is as safe as using a condom in preventing pregnancy.
6. When teenagers have sex for the first time, most of them use condoms.
7. Teenage men can seek advice from pregnancy counsellors.
8. What is the legal age of consent (the age at which you can legally first have sex) in the UK?

Attitudes

Male Role Attitudes Scale[5]

Responses to the 'Male Role Attitudes Scale' were based on a four-point Likert scale from 'strongly disagree=1' to 'strongly agree=4'. A total score was derived by totalling responses to each item, giving a possible score of 8-32, where higher scores indicate greater endorsement of traditional male role stereotypes.

1. It is essential for a man to get respect from others.
2. A man always deserves the respect of his wife/partner and children.
3. I admire a man who is sure of himself.
4. A man will lose respect if he talks about his problems.
5. A man should be physically tough, even if he is not big.
6. It bothers me when a man acts feminine/'like a girl'.
7. I don't think that a man should have to do housework.
8. Men are always ready for sex.

Skills

Comfort Communicating Scale[4]

Responses to the 'Comfort Communicating Scale' were based a four-point Likert scale from 'extremely uncomfortable=1' to 'extremely comfortable=4'. A total score was derived by totalling responses to all three questions, giving a possible score of 3-12 with higher scores indicating greater comfort.

How comfortable would you be…

1. Talking with a boyfriend/girlfriend about avoiding pregnancy
2. Talking with parents/carers about avoiding pregnancy
3. Talking with a health professional about avoiding pregnancy

Sexual Self-Efficacy Scale[6]

Responses to the 'Sexual Self-Efficacy Scale' were based on a four-point Likert scale from 'very difficult=1 to very easy=4. The score for this item was the mean response to all 13 items, calculated by totalling the responses to the 13 items and dividing this by 13, giving a possible score of 1-4, with higher scores indicating greater perceived self-efficacy.

In a sexual relationship now or in the future, how easy or difficult do you think it would be for you to

1. Tell your partner you don't want to have sex
2. Do something sexual (kissing with tongues, petting) with your partner without feeling you have to 'go all the way' (have sex)
3. Talk to your partner about using contraception (e.g. condoms or the pill)
4. Say no to having sex without contraception
5. Decide not to have sex if you are drunk or on drugs
6. Choose who you want to have sex with and when
7. Refuse to do something sexually (kissing with tongues, petting) which you don't feel comfortable about
8. Be able to get condoms/contraception
9. Talk to a doctor or other health professional about contraception

10. Talk about being sexually inexperienced to your sexually experienced peers
11. Say no to having sex with someone that you don't want to
12. Say no to having sex with someone who is drunk or on drugs
13. Wait until you are ready to have sex

Intentions

Intentions to avoid a teenage pregnancy scale (TUPS[7])

The response options for the 'TUPS' items were based on a five-point Likert scale, from 'very unlikely =1' to 'very likely=5'. An overall intentions score was derived by totalling responses to all items, giving a possible score of 12-60, where higher scores indicate stronger intentions.

When thinking about having sex, now or in the future, how likely is it that you would…

1. Be able to get contraception
2. Feel Okay about saying 'no' if you did not want to have sex
3. Be able to tell that both you and your partner were willing
4. Have sex at about the right time in a relationship
5. Know where to get advice about contraception
6. Think about the outcomes of an unintended pregnancy
7. Say no to sex unless you had contraception
8. Not have sex until you are ready
9. Make up your own mind when you are ready for sex
10. Talk to your partner about using contraception
11. Share the responsibility for using contraception with your partner
12. Resist any peer pressure to have sex

Overall, the reliability of the measures was satisfactory (see Table 1 below). The Intentions scale, Sexual Self-Efficacy scale, and Male Role Attitudes scale had high internal consistency (Cronbach's alpha in excess of 0.7). The Knowledge and Comfort Communicating scales had satisfactory internal consistency (Cronbach's alpha: 0.55–0.699).

Table 1 Instrument reliability and validity

**Scale**	**Cronbach's Alpha**
Knowledge	Scale reliability coefficient: 0·9396
Male Role Attitudes	Scale reliability coefficient: 0·9602
Comfort Communicating	Scale reliability coefficient: 0·9587
Sexual Self efficacy	Scale reliability coefficient: 0·9737
Intentions	Scale reliability coefficient: 0·9736

6. Process Evaluation Methods

Research design

The overall aim of the process evaluation was to understand implementation of the intervention as well as its acceptability and perceived effectiveness to pupils, teachers, parents and RSE stakeholders. The research design of the process evaluation was informed by realist approaches to the evaluation of interventions[8,9] as well as Medical Research Council (MRC) guidance[10] for process evaluations to consider the context; implementation; and mechanisms of impact of interventions.

Methods

Multiple methods were utilised with triangulated design. Table 2 provides an overview of the methods and data sources used in the process evaluation. All study schools were included in the process evaluation. In addition, more in-depth study was conducted in eight case study schools in the intervention arm. At the point of randomisation, the NICTU Trial Statistician randomly selected two schools in the intervention arm from each nation to act as case study schools.

Data Analysis

All audio files were transferred securely to QUB and transcribed verbatim (interviews and focus groups) or typed up by researchers (observational field notes and other secondary source data). Data were organised using NVivo 12 software and analysed systematically and thematically based on the six steps proposed by Braun and Clarke[11] to enable identification and analysis of patterns within the data by moving iteratively between theoretical understandings and the new data.

Methodological rigour was ensured by establishing credibility, transferability, dependability and confirmability using techniques suggested by Lincoln and Guba.[12] These inductively and deductively derived codes were first compiled as a code book and then applied to the data. These codes as applied to the data were discussed with the researchers from each of the four nations to check validity of emerging themes and elaborate complexity and deviancy in the data. All transcripts were independently coded by two members of the research team (10% coded by seven members). Meaningful quotes from participants were extracted to support and exemplify identified themes/sub-themes. Open-ended questions from the Student Engagement Questionnaire (SEQ) and parent survey were transferred to NVivo 12 and thematically coded as per other qualitative data.

Implementation fidelity data were entered into excel files in each site and transferred to QUB for summary descriptions. Data from the parents' survey and student engagement questionnaire were imported to Excel and SPSS and tabulated as summary statistics. Descriptive data analysis was conducted to identify general patterns in the data.

Table 2 **Process evaluation data collection: data sources**

School(s)	Data source	Key focus area/purpose	Data Collected
**All schools**	School Background Questionnaire	School structure, socio-demographic profiling, and experience of unintended teen pregnancy	58 schools (N=58)
	RSE Questionnaire	Extant RSE provision	29 intervention; 26 control schools) (N= 55)
	Fieldworker Perception Form	What worked well and what did not in relation to data collection; any other relevant observations mainly form lead field worker and some additionally form fieldworkers	Baseline: 66 schools<br>Follow-up: 60 schools (N=180)
**Intervention schools**	Parent/guardian online survey	Engagement with and opinion of the parent/guardian animations and homework session; whether their child discussed programme with them	N= 134
	Parent/guardian data analytics	Website viewing statistics of the parental component for the implementation period obtained using Google Analytics	N=1126 unique visits recorded
	Teacher Implementation Log	Recorded activities delivered in each lesson to assess each school's fidelity to programme guidance	29 schools N=128 implementation logs
	Semi-structured trial champion/principal/head teacher interviews	Perceived barriers and facilitators of successful implementation and engagement with different components of the intervention	29 individual interviews N=29<br>5 paired interviews N=10
	Student Engagement Questionnaire	Programme delivery, parental participation, quality of teaching and perceptions of their engagement in the programme	N=3,179
**Intervention case study schools [a]**	Lesson observations (Lead Fieldworker Observation Form)	Measuring teacher fidelity to implementation protocol and pupil engagement	8 schools N=35 observations
	Teacher focus groups	Perceived barriers and facilitators of successful implementation and engagement with different components of the intervention	8 focus groups N=31 participants
	Pupil focus groups	Perceived barriers and facilitators of successful implementation and engagement with different components of the intervention	8 focus groups N=58 participants
	Parent focus groups/paired interviews/interviews	Assess parental engagement and evaluation of the intervention	6 (5 case study schools; 1 non-case study school) N=10 participants
**External**	Education/policy specialist interviews	Current context of RSE policy and practices and perceptions of how this might influence programme uptake; acceptability of intervention and future implementation potential	N=11 interviews
	Audio/video-recordings of teacher training	Check fidelity of teacher training in randomly selected intervention schools	N=16 recordings
	Teacher trainer telephone interviews	Perceptions on quality of training materials and how training was received by teachers	N=8 interviews

[a] in addition to data collection outlined above in intervention schools

7. Sensitivity Analysis

Follow-up data was not available for four schools (3 intervention; 1 control). The sensitivity analyses made different assumptions on the best and worst case scenarios. The best-case scenario assumes that none of the students in the missing intervention schools had unprotected sex but 13% of students in control schools did. The worst-case scenario assumes that none of the students in the missing control school had unprotected sex but 13% of students in the intervention schools did. The rate of 13% was based on the school with highest rate of unprotected sex at baseline.

The results of the sensitivity analysis are presented in Table 3. In the adjusted best-case scenario, fewer students in the intervention school reported unprotected sex at 12-14 month follow up than those in control schools [12.05% vs 14.53%, OR 0.59 (95% CI 0.34 to 1.03), p=0.06]. In the adjusted worst-case scenario, a slightly higher number of students in the intervention schools reported unprotected sex than those in control schools [14.92% vs 13.49%, OR 1.39 (95% CI 0.71 to 2.71), p=0.34].

Table 3 Sensitivity analysis

	Intervention	Control	Adjusted OR (95% CI)[a] , *p*-value	ICC
**Primary Outcome**				
Best Case				
Yes, n (%)	425 (12·05%)	481 (14·53%)	0·59 (0·34, 1·03), p= 0·06	0·21
No, n (%)	3103 (88·0%)	2829 (85·5%)		
Worst Case				
Yes, n (%)	475 (14·92%)	463 (13·49%)	1·39 (0·71, 2·71), p= 0·34	0·31
No, n (%)	2709 (85·08%)	2968 (86·51%)		

[a] Adjusted for primary outcome at baseline, nation and above or below median FSM

An additional analysis based on risk ratios (RR) is provided as an additional robustness test to the reported odds ratios of the primary outcome and post-hoc exploratory analysis of the primary outcome component, 'use of reliable contraceptive at last sex'.

Primary outcome n=6260

OR: 0.85 (0.58 – 1.26) p=0.42

RR: 0.89 (0.65 – 1.22) p=0.47

Exploratory post-hoc analysis (use of reliable contraceptive) n=216

OR: 0.52(0.29 – 0.92) p=0.025

RR: 0.83 (0.70 – 0.98) p=0.025

8. Process Evaluation Results: RSE Provision

Current status of Relationship and Sexuality Education in the UK

Educational policy, including relationship and sexuality education is devolved to the four nations of the UK. Hence, the terminology to describe the education adolescents receive relating to sex and relationships varies in each nation as follows. Relationships and Sexuality Education (RSE) guidance in Northern Ireland is predominantly structured under the Personal Development strand of the Learning for Life and Work curriculum. In England, Sex and Relationship Education (SRE) is provided under the umbrella term Personal, Social, Health and Economic Education (PSHE). Pupils in Scotland learn about Relationships, Sexual Health and Parenthood Education (RSHP) as part of their Curriculum for Excellence framework. In Wales, Sex and Relationships Education (SRE) forms one of the six Areas of Learning Experience (AoLE). For the purposes of uniformity in this document, the appellation RSE is applied UK-wide. In England and Wales, RSE is currently undergoing significant reform. England introduced compulsory RSE in 2020/2021, post data collection for this trial[13] and Wales is set do to so in 2022.[14] In England, parents will still be able to opt-out their children when the topic of sex is being discussed. NI and Scotland have statutory guidance on what RSE should be taught, although this is not compulsory, and many schools follow their own guidance or ethos concerning how the curriculum is delivered[15,16]. Therefore, throughout the UK there is no uniform guidance for the curriculum and where guidance it exists, it is open to degrees of interpretation by schools in how it should be implemented.

What was the provision of RSE in control schools?

Control school RSE questionnaires were analysed to determine approach to RSE and identify any reported changes to provision which could have been due to participation in the *Jack Trial*. Responses were analysed using an assessment tool informed by the Whole School RSE Audit tool developed by the National Children's Bureau for the Sex Education Forum[17].

Were there changes to provision and potential contamination?

No schools reported changes to RSE provision as a result of participation in the trial. However, five of the control schools described ongoing attempts to develop and update provision, with some describing previous resources as ‘outdated’. More broadly, most schools reported at least some small changes to increase RSE provision in schools.. In general, there was an increased awareness in schools due to media coverage of sex education and increased awareness owing to external (State) guidance, namely the PSHE review in Scotland (2019) and the introduction of statutory RSE in England (2020). Overall, the control sites were not considered to be contaminated by changes to provision.

Comparison of Control and Intervention Schools

The same RSE provision questionnaire was used to assess RSE provision outside of *If I Were Jack* in both intervention and control schools. Results show that schools in both the control and intervention arms were broadly comparable/equal in terms of the distribution of schools categorised as having high, medium, or low provision of RSE, outside of *If I Were Jack* (see Table 4).

Table 4 The quality of RSE provision in Intervention and Control schools excluding provision of *If I Were Jack*

RSE in School Context	Number in Intervention Schools	Number in Control Schools
High provision	3	3
Medium provision	7	8
Low provision	19	15
Not available	5	6
**Total**	**32**	**32**

Summary

In summary, RSE provision in control schools was regarded as low to medium when judged against a tool designed to capture best practice. No schools reported changes to provision as a result of participation in the trial, but five schools reported updating their provision and most control schools made small ongoing changes to the provision of RSE.

9. Process Evaluation Results: Fidelity

Fidelity of implementation of teacher training delivery and fidelity of teacher-delivery of intervention in classroom

In all intervention schools, teacher trainers delivered training to teachers prior to programme implementation (n=13 teacher trainers: one in Scotland, two in NI, two in England, and nine in Wales). Training in Wales was delivered by the Healthy Schools coordinators (Public Health Wales staff). All teacher trainers were trained in a face-to-face two-hour session with the Jack Trial team prior to delivering teacher training.

A fidelity checklist was used to assess fidelity of teacher training. Each session was scored out of a possible maximum of 49 points and structured under nine sections to cover all aspects of the programme. Percentage scores were graded as <50% = low; 50-70% = medium; and >70% = high.

Table 5 Percentage of fidelity to teacher training protocol

Site	Number of trainers	Number of schools audio assessed	Avg % Fidelity	Fidelity Grading <50% = Low; 50-70% = Medium; >70% = High
N.I.	2	6	100%	High
England	2	4	61%	Medium
Scotland	1	4	99%	High
Wales	9	2	71%	High
TOTAL UK	14	16	83%	High

Fidelity of implementation in the classroom

Approximately 168 teachers delivered the resource in the 30 intervention schools that completed the trial across the UK. Classroom fidelity assessed through a teacher implementation log was scored out of a possible maximum of 13 points to cover all of the programme activities (Table 6). Teachers reported delivering an average of 83% of Intervention activities across all sites (see Table 5). Teachers in NI reported the highest delivery rate of all intervention materials (88%), followed by Wales (85%), England (80%) and Scotland (78%). Most activities were delivered at least 80% of the time. The IVD had the highest delivery rate (99%) and the parent survey the lowest (38%). Wallet cards, which signposted students to sources of sexual and reproductive health information and support services, had the next poorest delivery rate (75%). Independent researcher observations and interviews with teachers and students shed further light on implementation fidelity. While teacher creativity was most cited reason for a reduction in implementation fidelity, problems with information technology /computers was another and finally a perceived clash with school ethos was also cited. The latter was especially so in relation to the wallet cards of adolescent sexual and reproductive health services. Though not problematic overall, there were identified problems in two faith-based schools (a Catholic and Protestant school in different nations) with the student wallet card component. In particular, some teachers in these two schools felt the cards did not fit with the school ethos and were wary of issuing them in fear of complaints from parents. This trial was the first RSE trial of comprehensive RSE education in faith-based schools in the UK and there was no other clear pattern of failure to implement the intervention in faith-based schools.

Table 6 Percentage of activities delivered by nation (teacher reported)

Resource Activity	Nation				[a] Total UK
	NI	Eng	Scot	Wales	
Activity 1 – Intro (Not asked for in TIL)	---	---	---	---	---
Activity 2 - Ground rules	93%	88%	91%	100%	94%
Activity 3 – IVD	98%	100%	100%	100%	99%
Activity 4 – Pause, fast-forward, rewind	98%	88%	88%	89%	91%
Activity 5 – What about Emma?	95%	88%	72%	92%	87%
Activity 6 – If I had to look after a baby	88%	94%	75%	94%	87%
Activity 7 – Fact or Fiction?	88%	88%	75%	89%	85%
Activity 8 - Jack Wallet Card	84%	65%	53%	86%	75%
Activity 9 – Jack Forum Dilemmas	86%	82%	72%	92%	83%
Activity 10 – Online Scavenger Hunt	84%	59%	91%	94%	85%
Activity 11 – Homework Parent Survey	40%	53%	34%	33%	38%
Activity 12 – Staying Safe Scenarios	98%	76%	94%	92%	92%
Activity 13 – Controversial Statements	95%	82%	84%	89%	89%
Activity 14 – My Plan	95%	71%	91%	50%	78%
TOTAL ACTIVITIES DELIVERED	88%	80%	78%	85%	83%

[a] Average by nation and total percentages in this table calculated from individual class completion logs

Implementation fidelity of parental component

The parental component of the intervention was composed of two parts:

1. A URL link texted/emailed by the school to parents bringing them to a website with online animated films and material for parents/guardian on the topic and information for parents to tell them the intervention was happening in schools and opportunities for parents to participate with their child in the intervention.
2. A homework activity brought home by the student to do with a parent/guardian/trusted adult.

The results relating to parental engagement are reported in detail elsewhere[18]. A summary of the results are provided below.

Implementation fidelity for digital parental materials

The total potential parents/guardians that could have been reached was *circa* n=4,097. Website analytics indicated 1123 unique visits to ‘parent resources’ webpages during implementation, signifying around 27% of parents visited these on one occasion or more. Analytics showed that when visiting the website, 380 (9%) viewed the shorter animated film, 288 (7%) the longer animation, and 658 (16%) the interactive film excerpt.

Results from the online parent survey indicated that, of 134 respondents, 50% had watched the short animated ‘hook’ feature, 45% the longer animated instructional feature, and 42% the interactive film excerpt. While 4% of respondents said they did not watch the films because they were not interested; most said they did not know about them (68%), forgot (14%), or did not have time (11%).

Implementation fidelity for the parent-student homework communication exercise

The above online digital materials were intended to prepare parents for an optional parent-student homework exercise assigned by teachers. Teachers were instructed to assign the homework exercise to students, informing them that, while they recommended they complete the exercise, it was not compulsory.

Across the four sites, an implementation rate of 38% was reported in teacher implementation logs for the homework exercise, ranging from 33% in Wales, to 53% in England. A similar figure of 34% of parent survey respondents said they had completed the exercise with their child. However, the student engagement questionnaire responses (n=3179) revealed that only 13% of students (n=403) said they had completed the task with their parents. Student-reported implementation varied by nation, with 17% in NI, 11% in England and Wales, and 10% in Scotland. Differences in completion rates by sex of student was evident with an independent t-test showing significantly more females reporting completion than males $t(2955)=2.5$, $p=0.011$.

10. Economic Evaluation

Aim

The aim of the economic evaluation was to describe the costs and consequences of implementing *If I were Jack* in UK schools to provide information to decision makers on the implications of rolling out the intervention further. Two analyses were conducted:

1) *Within-trial economic analysis*. The aim of this analysis was to calculate the cost of teacher time to deliver *If I Were Jack* compared to existing Relationships and Sex Education (RSE) provision (and hence the opportunity cost) based on teacher completed resource use questionnaires. Descriptive statistics for sexual health related health care resource use and costs and inputs to the model based on participant completed data were also calculated.
2) *Decision analytic model*. The aim of this analysis was to report the long term cost-effectiveness of *If I Were Jack* based on a decision model with input data collected from the trial in addition to relevant data from the published literature[19,20].

Methods

Within-trial analysis

Descriptive statistics for the percentage of students and mean number of contacts for self-reported health service use were reported for students in the control and intervention arm. Resource use for each student was multiplied by unit costs from published sources (see Table 7). Both unadjusted and adjusted mean cost per student at follow-up were reported for the control and intervention groups, the latter obtained after adjusting for baseline resource use and stratification variables using clustering as a random effect. 95% CIs were calculated based on bootstrapped bias corrected methods.

Table 7. Unit costs sexual health resource use

Resource	Cost(£)	Reference
Sexual health consultation in clinic	£120	PSSRU[20]
Sexual health advice from school nurse	£11	PSSRU
Sexual health advice from GP	£34	PSSRU
Sexual health advice from GP nurse	£12	NICE[21]
Condom (assumed three condoms per pack)	£2	Boots[22]
Other contraception (average cost of different contraceptives indicated by the students in the trial)	£7.16	Boots
Pregnancy test	£4	Boots
STI test	£16.50	NICE
Emergency contraception	£30	Boots

Long term decision model

We published a behaviour change-based decision model[23] aimed at assessing the long term cost-effectiveness of the *If I Were Jack* versus existing RSE. The model was populated using evidence collected from the trial and published literature on different types of outcomes which are directly and indirectly related to pregnancies and STIs among young people. Where no trial data were available, assumptions were made from pre-existing data and expert input. The model had a 20 year time horizon and calculated the expected costs and consequences for a hypothetical cohort of patients with similar characteristics to those enrolled in the trial. Both deterministic and probabilistic sensitivity analysis were conducted to assess the robustness of the results to alternative modelling assumptions. The long-term decision analysis model was conducted in R version 4.0.3.

Design

A conceptual model developed to evaluate the long term cost-effectiveness of the intervention, which incorporated theories of behaviour change is illustrated in Figure 2.

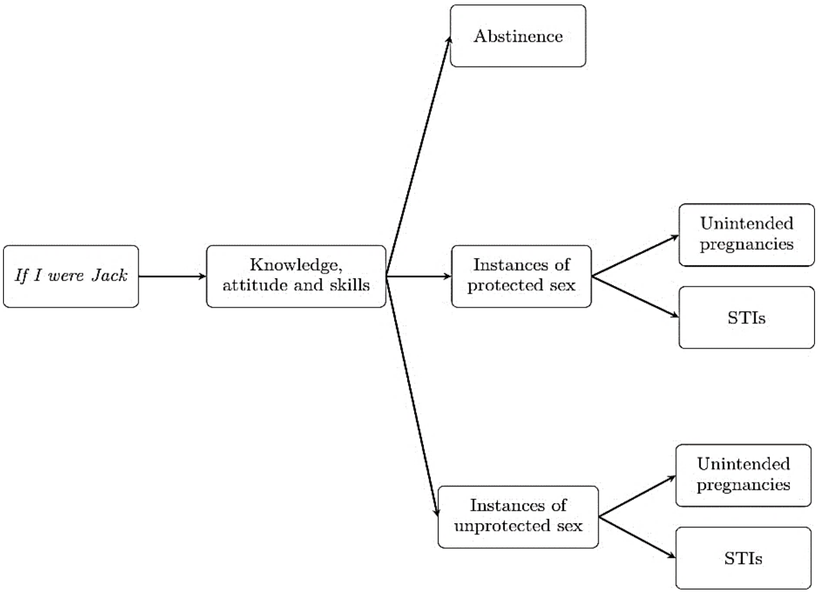

Figure 2. Conceptual model of the *If I were Jack* intervention

Reading from left to right, we started with the target population (young people aged <16) who engaged with the *If I were Jack* intervention and experience an increase in the levels of the cognitive outcomes in topics related to sexual health (knowledge, attitude and skills). We hypothesised a change in the behaviour outcomes with an increase in the number of people who delayed vaginal sexual intercourse (abstinence), an increase in the instances of protected sex for those sexually active, and a decrease in the instances of unprotected sex. This, in turn, would lead to a decrease in the number of unintended pregnancies and STIs in the target population.

Population. The target population was young people in post-primary schools in the UK age circa 14 years years of age within the general population. An intervention that encourages the use of contraceptives is unlikely to prevent young people who hoped to become pregnant from endeavouring to become pregnant. The model therefore assumes that the intervention is aimed at adolescents who want to prevent unintended pregnancy.

Outcomes. The outcomes were reported in terms of the output of a cost-benefit or return on investment (ROI) analysis, whereby both costs and benefits are measured in monetary units. The "benefits" were the cost savings that result from averted pregnancies. The "cost" was the total amount of public money currently being spent for preventing pregnancies and STIs among young people.

Perspective. The analysis took a public sector perspective which included costs incurred by the public sector. The analysis was presented with and without the inclusion of government-funded Benefits. We reported aggregated costs by public sector (education, health-care, welfare).

Modelling the health consequences of behavioural change

An extensive literature search and consultation with experts was conducted to identify quantitative evidence about the different elements (cognitive, behavioural and biological) of the theory of change behaviour that could be used to inform the final structure of the model for the target population. However, since no evidence was found about how variations in the cognitive outcomes could be converted into changes in the behavioural and biological outcomes, the structure of the model shown in Figure 2 was modified and focussed on how changes in behavioural outcomes were translated into changes in biological outcomes. Specifically, the updated model assumed that the behavioural change targeted by the intervention will result in a delay in sexual debut and/or increase of contraception use. The analysis focussed on how any changes in these behavioural outcomes affected the number of pregnancies and cases of STIs.

Modelling the number of pregnancies

To estimate the number of pregnancies averted, we used a mathematical model[24,25] to define how different types of contraceptive methods were translated into cases of pregnancy.

$$Y = N_f\left(g_{con}K_{con} + g_{pil}K_{pil} + g_{eme}K_{eme} + g_{inj}K_{inj} + g_{dia}K_{dia} + g_{imp}K_{imp} + g_{iud}K_{iud} + g_{wit}K_{wit} + g_{noc}L\right).$$

The total number of pregnancies (Y) depended on the number of sexually active females in each arm of the study (N_f), the probability of becoming pregnant within one year without contraception (L), the percentage of students using condoms (g_{con}), pill/patch/ring (g_{pil}), emergency pill (g_{eme}), injection (g_{inj}), diaphragm/cap/spermicide (g_{dia}), implant (g_{imp}), intrauterine device (g_{iud}), withdrawal (g_{wit}) or no contraception (g_{noc}), and the contraceptive failure rates for each contraceptive class (K_{con}, K_{pil}, K_{eme}, K_{inj}, K_{dia}, K_{imp}, K_{iud}, K_{wit}). Evidence on the failure rates was taken from the return-on-investment analysis of improving access to contraception in the UK[26]. The failure rate was defined as the percentage of women experiencing unintended pregnancy within one year of typical use of that contraceptive method. The rates of contraception use were taken from responses to the follow-up questionnaire in the *If I were Jack* trial. The impact that social disadvantage may have on the number of unplanned pregnancies was accounted for based on evidence from the third National Survey of Sexual Attitudes and Lifestyles[27] and the proportion of pupils receiving free school meals collected from the trial[28]. Sensitivity analysis was conducted to assess the impact that variations in the area-level deprivation effect and failure rates of the contraceptive methods may have on the final results.

The proportion of sexually active males and females aged less than 16-years old was estimated using the answers to the Health Behaviour in School-Aged Children (HBSC) surveys for England[28], Wales[29] and Scotland[30] and the Young Life and Times[31] survey for Northern Ireland. We took this frequency to be an approximation of the proportion of sexually active females and males in the target population in each country. We used National Office of Statistics (NOS) data about the population estimates of females and number of births by age in 2017 in England, Wales, Scotland and Northern Ireland to obtain the total number of sexually active females in the target population in each country[32–37]. We then combined these data with the evidence from the answers to the NATSAL-3 survey to obtain estimates of the number of sexually active females in three different age groups: 11-13, 14 and 15 years.

There were no directly measured data for the probability of becoming pregnant within one year without contraceptive use in the UK. For the US, evidence from the published literature was available to first calculate the one-month pregnancy probability and convert this into a one-year probability[25]. This evidence was adapted in the literature using data from about the HBSC surveys for England, Wales and Scotland and the 2011 YLT survey for Northern Ireland. We used the available evidence on the conceptions and population of females, baseline condom use and annual condom failure rate to derive an estimate of L for each country in the UK among the young people aged less than 16-years old[24,28–37]. We included in the model different types of outcomes associated with the number of pregnancies averted using the structure shown in Figure 3.

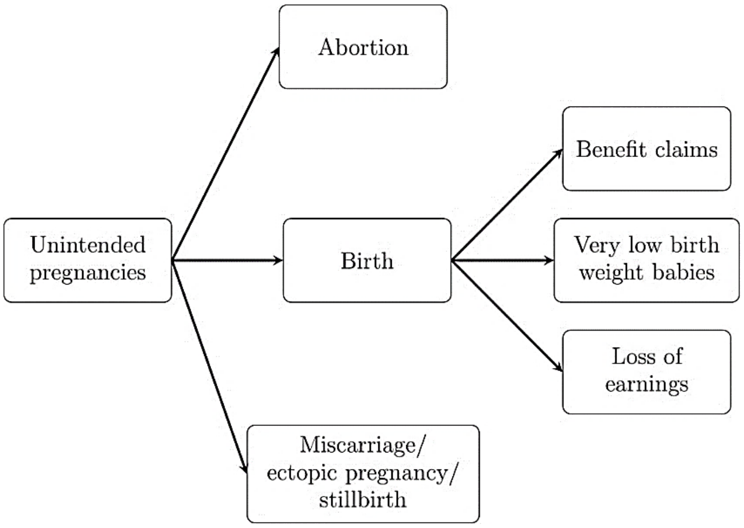

Figure 3. Model structure for the outcome associated with the number of pregnancies averted

The probability of becoming pregnant in the target population was expressed, following conception, in terms of the probability of females giving birth, terminating the pregnancy, miscarrying, having an ectopic pregnancy or a stillbirth. The probability of birth, abortion, miscarriage/ectopic pregnancy/stillbirth was based upon national government statistics for England, Wales and Scotland[32,33,35]. For Northern Ireland, the model uses the information about the number of abortions in England and Wales for Northern Ireland residents[34]. The probability of miscarriage/ectopic pregnancy/stillbirth is reported by Hospital Episode Statistics (HES) data collected by the Information Centre for Health and Social Care[34].

Modelling the number of STIs

The number of infections of each type of STI for each individual were modelled using a discrete event simulation approach based on an individual (or agent) multi-STI model[38]. The model estimated the number of STI cases averted for HIV and also for chlamydia, gonorrhoea and genital warts, according to the risk of infection as shown above and the proportion of sexually active individuals who received the intervention.

Sexual Behaviour

Evidence about the frequency of vaginal sexual intercourse, number of sexual partners and proportions of sexually active teenagers using condoms for vaginal sexual intercourse was taken from the trial. Evidence about the effectiveness of condoms for protecting against STI infections and reducing heterosexual HIV transmission was estimated based on evidence from the literature[39–46]. For genital warts, we used the evidence from a longitudinal study on young people[47], while a Cochrane systematic review estimated the reduction in HIV incidence due to condom use[48].

Benefit and cost outcomes

Monetising quality of life and other non-cost benefits

Utility scores were not assigned to teenage conceptions due to lack of evidence from the literature, difficulty in reaching a consensus around the valuation of pregnancies carried to term and those that are not, and the fact that it is not by any means certain that pregnancy as a health state generally diminishes health related quality of life[49,50]. However, the model can explore the cost-effectiveness of reduced young people conceptions based on willingness to pay criteria. The utilities associated with various STI outcomes were estimated using the evidence from the published literature[51–53]. We used these values to assign the utility weights to each type of STI considered, which were then used to calculate the quality adjusted life years (QALYs) gained from the reduction in cases of chlamydia, gonorrhoea, genital warts and HIV. The QALYs gained from reduction in each STI was calculated by multiplying the cases averted by (1 – utility for STI) multiplied by (1- proportion treated for STI).

Costs

The costs incorporated within the health economic model included:

- The costs from the trial for the intervention and control group
- Contraception methods and maternity costs
- Abortion and Miscarriage costs
- Costs for the treatment of very low birth weight babies
- Costs for the testing and treatment of STIs and their health consequences
- Government-funded Benefits
- Medium and long-term loss of earnings due to teenage pregnancy

The costs associated with the resource use collected in the trial are described in section 2 above. The average costs of birth, abortion and miscarriage/ectopic pregnancy/stillbirth were taken from the NHS reference costs[54]. The cost of pre-school and primary school education for children was also considered in the analysis, under the assumption that the government would maintain funding at current levels[55]. While we recognise there are variations in school age start and costs across the four nations, the figure for England was used. The mean per pupil funding was taken from the analysis of the Institute for Fiscal Studies (IFS), with preschool costs only incurred at age 3 and 4, while primary school costs were incurred from age 5[56]. The cost of treatment of STIs was based upon expert guidance and the health economic model developed for the NICE Sex and Relationship Education (SRE) public health guidance[57]. Treatment for chlamydia is assumed to require Doxycyline 100mgs twice a day for 1 week and Azithromycin 1g followed by 500mgs daily for next 2 days. Given that a lot of first line treatment for Chlamydia is done outside specialist care, the models assumes that treatment is done for 50% of the times in GP practices and for 50% in GUM clinics. A sensitivity analysis to assess the impact on the results of assuming that treatment for Chlamydia is done always in GUM clinics is also explored. Treatment for Gonorrhoea is assumed to require Ceftriaxone 1g IM injection in GUM clinics, whilst treatment for genital warts is assumed to require one dose of imiquimod and a GP consultation. An average cost of HIV treatment per year was estimated based upon the sum of the average cost of care for HIV patients and the average cost of drug treatment. The costs of Benefit payments were also included within the model as they were costs incurred by the public sector, which represent real resource savings if the child that might otherwise eventuate had not been conceived. Evidence on different types of Benefit payments (Child Benefits, Child Tax Credits, Income Support and Housing Benefits) for the target population was taken from published sources, such as the Teenage Pregnancy Strategy report[58] and governmental sources[59,60].

Finally, when calculating the number of pregnancies and births averted through the use of contraception, it is generally not correct to assume that all of these births, if avoided today, would not have occurred later as a planned birth[57]. Evidence from the USA National Survey of Family Growth suggests that 60% of unplanned births are mistimed, whereas 40% of births would have otherwise never occurred[61]. The full direct and indirect costs of birth can only be considered for those 40% of unplanned births that would have not occurred later. For the remaining 60% of births which are mistimed, the cost averted by contraception was the cost of incurring expenditure at an earlier point than otherwise would have occurred later. We calculated the cost of a mistimed birth (MB) as

$$\mathrm{MB} = \mathrm{B} - \frac{\mathrm{B}}{(1+\mathrm{r})^{\mathrm{d}}}$$

where B is the cost of a birth, r the discount rate and d is the number of years by which the birth would have been delayed (taken as 2 years in the literature[62]). Given that the proportion of mistimed births was based on US data, we varied this in sensitivity analysis to assess its impact on the results.

Sensitivity analysis

A number of deterministic (DSA) and probabilistic (PSA) sensitivity analyses were undertaken to assess the impact on the results of key parameters and assumptions in the model. Under each scenario, each parameter was varied either separately in a deterministic way (DSA) or simultaneously using probability distributions (PSA) over a large number of iterations of the model.

Cost-effectiveness analysis

We calculated the mean incremental total cost of *If I Were Jack* compared to standard RSE practice for (i) cost per pregnancy averted; (ii) cost per STI averted; (iii) cost per QALY gained over a 20 year time under a healthcare (excluding government-funded Benefits) and public sector perspective (including government-funded Benefits).

All benefits and costs after 12 months were discounted at an annual rate of 3.5% in line with NICE guidance to capture time preferences for costs and benefits[63]. All costs are reported in 2019/2020 British Pounds.

Results

The cost of teacher training and teacher time delivering If I Were Jack

The cost of *If I Were Jack* was based on the opportunity cost of teachers delivering the intervention. The data were collected via questionnaires from a total of 96 teachers across 30 intervention schools. Estimates for the lesson duration were obtained by taking the average time required for each type of lesson: type A (50-60 min), type B (6 35-45 min), type C (drop-down day/two half days). Estimates for the overall time spent and associated cost[19] were then derived by summing up the estimates computed for each lesson type and dividing by the total number of students in the *If I Were Jack* group (Table 8).For comparison standard RSE costs £4.42 per student.

Table 8 Teachers' resource use and costs for preparing and delivering the *If I were Jack* intervention. Summary statistics are reported in terms of number and proportion of observations, means and standard deviations.

**Resource use**	**N (%)**	**Time (min) – mean (sd)**	**Costs (£) – mean (sd)**
Lesson preparation	87 (0.91)	109 (84)	£44 (£34)
Photocopy preparation	63 (0.66)	36 (51)	£35 (£82)
Other input (e.g. discussion)	63 (0.66)	78 (114)	£31 (£45)
Other input (e.g. equipment)	50 (0.52)	-	£77 (£74)
Overall preparation	92 (0.96)	187 (172)	£139 (£133)
Lesson delivery	94 (0.98)	262 (89)	£104 (£36)
**Total**	94 (0.98)	438 (229)	£240 (£153)
**Treatment cost per student**	-	-	£5.48

Sexual health resource use and costs

Each type of sexual health resource use was summarised in terms of number and proportion of observations, means and standard deviations (see Table 9). Given the large number of students who did not use any resource, summary statistics related to the resource use questionnaire data were reported for students that used the resource only. Cost data were summarised in terms of means and standard deviations for all students since the average costs in the economic evaluation should be calculated based on all participants (see Table 10). The mean adjusted difference of *If I Were Jack* minus standard RSE for healthcare resource use costs only is -£2.33 (95% CI -£6.73 to £2.07) per student, adjusting for stratification variables (proportion of free school meals and country) using a random intercept model to account for clustering (students within schools).

Table 9 Number of students with percentage that used each type of resource and mean (sd) resource use for those that used the resource

**Resource**	**Baseline**		**Follow-up**	
	**Standard RSE N=4100**	**Jack N=4116**	**Standard RSE N=3178**	**Jack N=3341**
**Sexual health clinic**				
n (%)	58 (0.014)	57 (0.014)	150 (0.047)	113 (0.034)
Mean (sd)	1.67 (1.05)	2.29 (2.53)	2.11 (1.70)	2.07 (1.82)
**School nurse**				
n (%)	256 (0.062)	271 (0.066)	105 (0.033)	131 (0.039)
Mean (sd)	1.57 (1.05)	1.61 (0.93)	1.8 (1.24)	1.63 (1.06)
**GP**				
n (%)	62 (0.015)	81 (0.020)	138 (0.043)	141 (0.042)
Mean (sd)	1.53 (0.95)	1.81 (1.64)	1.67 (1.34)	1.38 (0.80)
**GP nurse**				
n (%)	40 (0.010)	41 (0.010)	55 (0.017)	79 (0.024)
Mean (sd)	1.25 (0.54)	1.80 (1.71)	1.64 (1.22)	1.71 (2.05)
**Free condoms**				
n (%)	256 (0.062)	395 (0.096)	434 (0.137)	427 (0.128)
Mean (sd)	2.56 (4.67)	3.29 (5.31)	2.93 (3.96)	3.26 (4.67)
**Bought condoms**				
n (%)	103 (0.025)	155 (0.038)	299 (0.094)	395 (0.103)
Mean (sd)	4.17 (7.24)	3.92 (7.87)	3.35 (4.81)	3.38 (4.80)
**Other contraception**				
n (%)	53 (0.013)	75 (0.018)	214 (0.067)	218 (0.065)
Mean (sd)	1.77 (1.92)	1.75 (1.44)	2.03 (2.13)	2.12 (2.28)
**Pregnancy test**				
n (%)	33 (0.008)	50 (0.012)	85 (0.027)	87 (0.026)
Mean (sd)	1.67 (1.08)	1.30 (0.84)	1.48 (0.87)	2.05 (3.14)
**STI test**				
n (%)	23 (0.006)	33 (0.008)	50 (0.015)	55 (0.016)
Mean (sd)	1.52 (0.99)	2.55 (3.09)	1.50 (0.84)	2.22 (3.60)
**Emergency contraception**				
n (%)	18 (0.004)	25 (0.006)	78 (0.025)	96 (0.029)
Mean (sd)	2.17 (2.96)	1.76 (2.24)	1.31 (0.84)	1.98 (2.35)

Table 10 Sexual health care resource use: observed cost means and standard deviations (£) per participant for all students.

	Baseline		Follow-Up	
	**Standard RSE Mean(sd)**	**Jack Mean(sd)**	**Standard RSE Mean(sd)**	**Jack Mean(sd)**
**Sexual health clinic**	2.992 (28.708)	4.030 (49.163)	12.352 (70.706)	8.764 (61.427)
**School nurse**	1.146 (5.225)	1.240 (5.264)	0.681 (4.404)	0.734 (4.260)
**GP**	0.832 (7.692)	1.284 (11.884)	2.552 (15.207)	2.054 (11.135)
**GP nurse**	0.154 (1.649)	0.228 (3.033)	0.353 (3.256)	0.505 (4.987)
**Free condoms**	0.336 (2.704)	0.665 (3.914)	0.833 (3.616)	0.869 (4.063)
**Bought condoms**	0.219 (2.692)	0.310 (3.474)	0.657 (3.607)	0.725 (3.774)
**Other contraception**	0.173 (2.163)	0.241 (2.228)	1.019 (5.485)	1.035 (5.726)
**Pregnancy test**	0.056 (0.725)	0.066 (0.695)	0.164 (1.130)	0.221 (2.443)
**STI test**	0.148 (2.284)	0.354 (6.001)	0.403 (3.587)	0.623 (9.029)
**Emergency contraception**	0.299 (7.320)	0.338 (6.744)	0.997 (7.360)	1.765 (15.742)
**Total costs**	**Mean(sd) [95%CI]**	**Mean(sd) [95%CI]**	**Mean(sd) [95%CI]**	**Mean(sd) [95%CI]**
	5.485 (33.979) [4.303;6.667]	7.403 (59.531) [5.384;9.422]	18.329 (78.078) [15.614;21.045]	14.090 (63.544) [11.935;16.246]

Within-trial total costs

For each student, the total costs were computed as the sum of the costs of delivering the *If I were Jack* intervention, obtained from the teacher completed resource use questionnaires, and the costs of the health care resource use, obtained from the student completed questionnaires. The total mean incremental cost of the Jack trial intervention compared to standard RSE was £2.83 (95% CI -£2.64 to £8.29) per student. The adjusted mean total cost per student at follow-up obtained from the complete cases was £22.59 (95% CI £16.27 to £28.88) for the Jack intervention and £19.78 (95% CI £13.59 to £25.96) for standard RSE. The adjusted analysis controlled for baseline total costs and stratification variables (proportion of FSM and nation) using a random intercept model to account for clustering (students within schools). 95% CIs were calculated based on bootstrapped bias corrected methods over a total of 10000 iterations.

For the MICE sensitivity analysis with 20 imputed datasets, the total mean incremental cost per student of *If I were Jack* compared to standard RSE was £1.81 (95% CI -£7.67 to £4.04).

We also included information on the cost of adapting the intervention to different groups, or updating it over time. The direct costs of making an adapted version for England/Wales with new video production was £35,000 to include all interactive video production and production of adapted online resource materials. Divided by the number of young people randomised to the intervention in England (n=1071) and Wales (n=1160) the cost per student was £15.69, which represents the upper estimate of the cost per student to adapt the intervention. If the intervention were rolled out to all young people 14 years of age in England (664,025 in 2020) and Wales (35,967 in 2020) the cost per student would be £0.05. The direct costs of making changes to the online resource materials for Scotland was £1000 or £1.15 per student if divided by the number of students randomised to the intervention in Scotland (n=866) or £0.02 per student if rolled out to all students aged 14 in Scotland (57,487 in 2020).

Consequences and decision model inputs

Summary statistics for different types of consequences (by type of contraception and STI) at follow-up are reported in Table 11.

Table 11 Summary statistics for consequences at follow-up in the standard RSE and Jack group for students defined as ever having sex.

**Consequences – n (%)**	**Standard RSE N=688**	**Jack N=733**	**All N=1472**
**Type of contraception**			
Pill	188 (0.273)	193 (0.263)	381 (0.268)
Condom	380 (0.552)	402 (0.548)	782 (0.550)
Emergency pill	34 (0.049)	21 (0.029)	55 (0.039)
Injection	32 (0.047)	11 (0.015)	43 (0.030)
Implant	57 (0.083)	39 (0.053)	96 (0.068)
IUD	6 (0.009)	4 (0.005)	10 (0.007)
Diaphragm/cap/spermicide	1 (0.001)	1 (0.001)	2 (0.001)
Other	6 (0.009)	4 (0.005)	10 (0.007)
None	126 (0.183)	164 (0.224)	290 (0.204)
**Type of STI**			
Chlamydia	3 (0.004)	11 (0.015)	14 (0.010)
Genital warts	3 (0.004)	5 (0.007)	8 (0.006)
Herpes	2 (0.003)	7 (0.010)	9 (0.006)
Gonorrhoea	5 (0.007)	7 (0.010)	12 (0.008)
Pubic lice	2 (0.003)	7 (0.010)	9 (0.006)
Trichomonas	2 (0.003)	5 (0.007)	7 (0.005)
Syphilis	4 (0.006)	7 (0.010)	11 (0.008)
HIV	3 (0.004)	7 (0.010)	10 (0.007)
Hepatitis	2 (0.003)	6 (0.008)	8 (0.006)
PID	2 (0.003)	5 (0.007)	7 (0.006)
Other	2(0.003)	4 (0.005)	6 (0.004)

Long term decision model

Over 20 years, per 100,000 young people receiving *If I were Jack*, compared to standard RSE Jack resulted in 379 fewer unintended pregnancies, 680 fewer STIs, 10 additional QALYs and at a cost saving of £989,406 (excluding government-funded benefits), or a cost saving of £9.89 per young person who received Jack. The largest gap was observed in the number of unintended pregnancies that resulted in abortions (1447 for RSE vs 1229 in Jack), followed by births (644 in RSE vs 559 in Jack) and miscarriages (272 in RSE vs 220 in Jack). Similar trends were observed for most types of STIs, with the number of chlamydia cases being associated with the largest discrepancy between the two interventions (1090 in RSE vs 635 in Jack), followed by genital warts (363 in RSE vs 227 in Jack). Relatively small differences were observed in the cases of gonorrhoea (165 in RSE and 153 in Jack) and PID (females only) (94 in RSE and 80 in Jack), while no substantial difference was observed in the number of HIV (for both groups around 1). There were no differences between the intervention and control groups in relation to frequency of sexual intercourse in those participants who were sexually active between baseline and follow up [aOR 0.99 (95% CI 0.76 to 1.29), p=0.93].

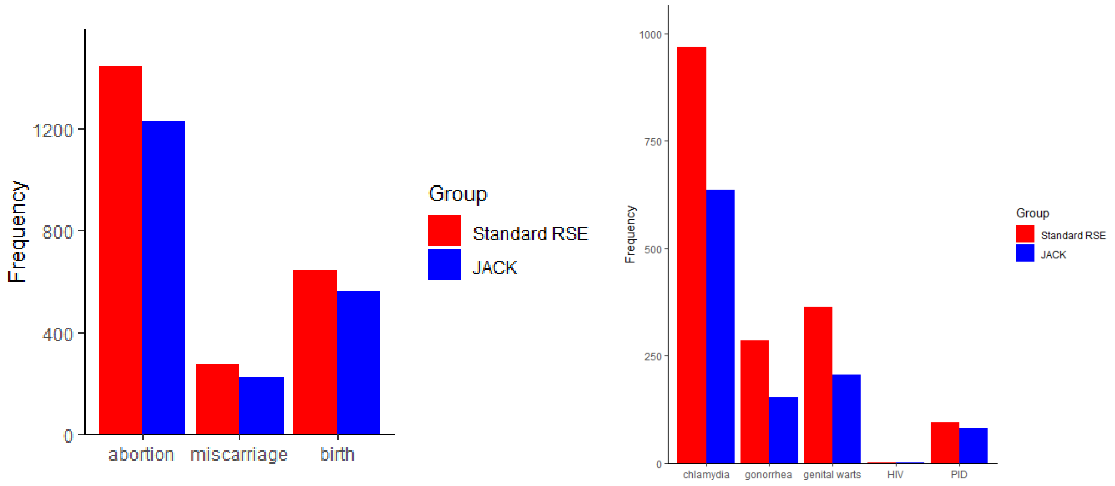

Figure 4 Total number of teenage (<16 years) unintended pregnancies and STIs per 100,000 young people over a time horizon of 20 years for standard RSE compared to the Jack intervention.

Probabilistic sensitivity analysis

Table 12-13 report the results from the probabilistic sensitivity analysis (PSA) in which the model parameters were randomly varied by drawing their values from associated probability distributions.

Incremental results obtained from the PSA, quantifying the impact of sampling uncertainty on basecase estimates, are reported in Figure 5 using separate cost-effectiveness planes for each type of averted outcome. The majority of iterations (97.5% for pregnancies, 96.6% for STIs and 95.7% for QALYs) were located in the South-East quadrant as the *If I Were Jack* intervention resulted in better outcomes for a lower cost.

Table 12 Estimates (from basecase) and 95% bootstrapped confidence intervals (from the probabilistic sensitivity analysis) based on 1000 iterations, including the number of outcomes (unintended pregnancies, STIs or QALYs) and total costs associated with each group as well as the averted outcome and incremental cost results between the Jack and standard RSE intervention. Costs were calculated either excluding (without) or including (with) government-funded benefits.

	**n - 95% CI**		**Total Costs (without benefits) – 95% CI**		**Total costs (with benefits) – 95% CI**	
	**RSE**	**Jack**	**RSE**	**Jack**	**RSE**	**Jack**
**Unintended pregnancies**	2,531 (2,461;3,100)	2,152 (2,078;2,716)	£20,459,742 (£18,687,125; £24,591,145)	£19,470,336 (£17,003,319; £22,175,576)	£191,457,170 (£140,548,595;£ 217,430,076)	£166,111,565 (£134,884,491;£ 171,019,049)
**STIs**	1,853 (1,106;3,400)	1,173 (412;2,467)				
**QALYs loss**	28 (19;45)	18 (14;30)				
**Averted outcomes/Incremental costs**						
**Unintended pregnancies**	379 (231;477)		-£989,406 (-£1,559,545;-£382,949)		-£25,345,605 (-47,857,741;- £5,438,251)	
**STIs**	680 (189;1,467)					
**QALYs**	10 (5;16)					

Table 13. Cost estimates (from basecase) and 95% bootstrapped CI (from the PSA) based on 1000 iterations) from the decision model disaggregated into seven components: contraception, pregnancy, abortion, miscarriage/ectopic pregnancy/stillbirth, STI treatment, birth, government-funded benefits. For each component, discounted cost estimates associated with standard RSE, Jack and the incremental results are displayed.

	**Standard RSE**<br>Estimate<br>(95%CI)	**Jack**<br>Estimate<br>(95%CI)	**Incremental**<br>Estimate<br>(95%CI)
**Discounted**			
**Contraception costs**	£4,832,457<br>(£4,645,046; £5,045,220)	£5,982,093<br>(5,791,743;6,190,293)	£1,145,703<br>(1,126,841;1,216,578)
**Pregnancy costs**	£600,064<br>(506,366;716,344)	£596,131<br>(480,238;703,678)	-£3,933<br>(-50,181;42,480)
**Abortion costs**	£987,846<br>(825,467;1,139,093)	£842,759<br>(747,888;947,512)	-£145,087<br>(-214,646;-60,854)
**Miscarriage/EP/ Stillbirth costs**	£99,396<br>(73,811;141,232)	£81,888<br>(54,695;104,823)	-£17,507<br>(-36,428;-2,020)
**STI treatment**	£270,122<br>(149,359;693,619)	£ 172,520<br>(132,954;486,693)	-£97,602<br>(-207,776;150)
**Birth costs**	£13,885,602<br>(11,576,613;16,041,941)	£11,928,685<br>(9,454,455;13,888,264)	-£1,956,917<br>(-3,299,170;-413,975)
**Government-funded benefits***	£170,997,429<br>(156,208,678;183,834,114)	£146,641,228<br>(140,286,029;153,431,931)	-£24,356,201<br>(-32,711,305;-15,922,648)

*****These costs were calculated by adding up the costs associated with the application and obtainment of government-funded benefits for young mothers as well as state costs aimed at supporting the growth of their children. These costs included: costs for child healthcare, costs of child school education, costs of child benefit, costs of child tax credit, costs of housing benefits, costs of income support.

PSA: Cost-Effectiveness Planes

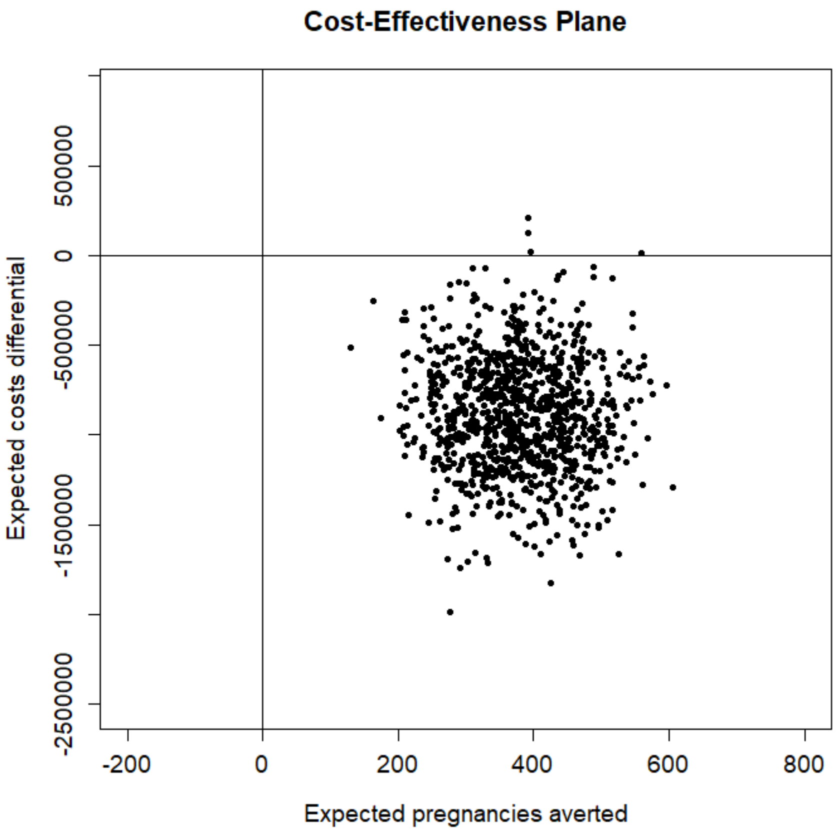

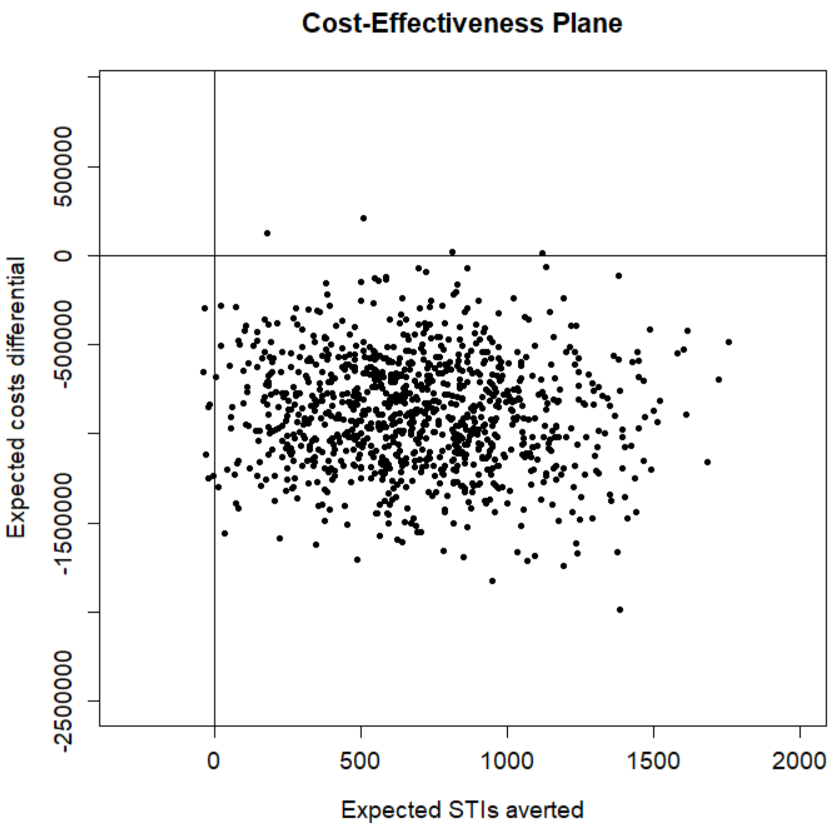

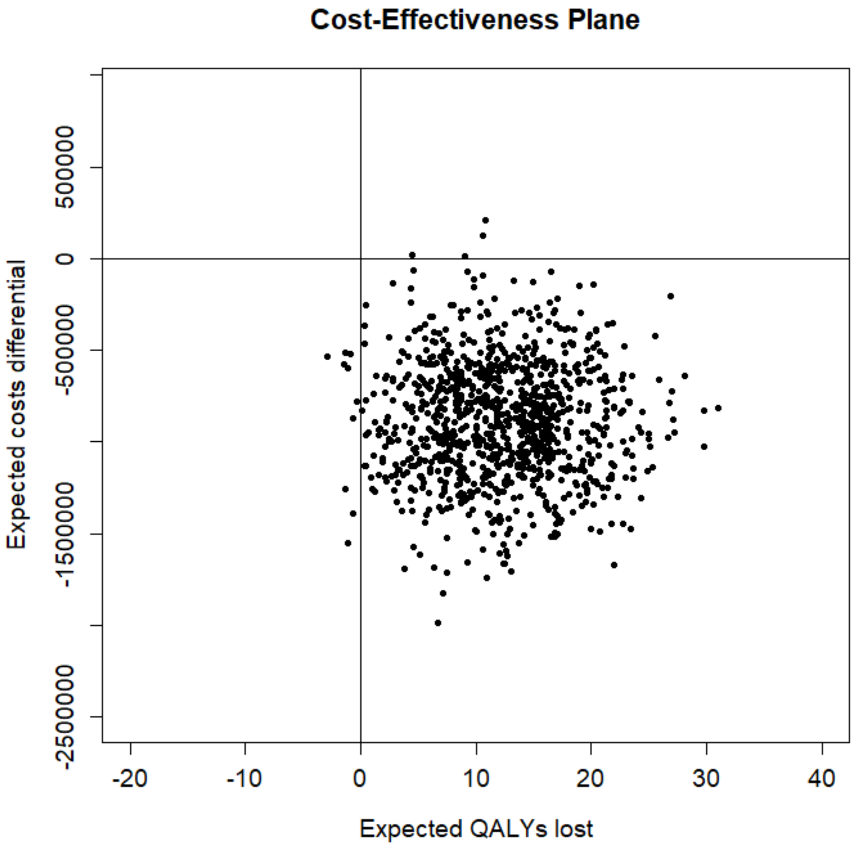

Figure 5 Cost effectiveness planes for the expected number of averted outcomes (unintended pregnancies, STIs and QALY losses) and the total costs increment (without state benefits) between the two intervention groups. Estimates are obtained based on a total of 1000 iterations for the PSA.

Deterministic sensitivity analysis

The results of the one- and two-way sensitivity analyses testing low and high extremes in values for failure rates (the probability of an unintended pregnancy) for condoms and withdrawal. In all scenarios the *If I were Jack* intervention exceeded standard RSE in that it resulted in better outcomes for a lower cost, except under the extreme scenario where the failure rate of condoms was halved (from 18% in basecase to 9%), with a total number of 178 averted pregnancies (compared to the 379 in the basecase) and an incremental total cost of £1,131,487 (excluding Benefits).

References

1 Hoffmann TC, Glasziou PP, Boutron I, *et al.* Better reporting of interventions: Template for intervention description and replication (TIDieR) checklist and guide. *BMJ (Online)* 2014; **348**. DOI:10.1136/bmj.g1687.

2 Lohan M, Aventin Á, Clarke M, *et al.* Can Teenage Men Be Targeted to Prevent Teenage Pregnancy? A Feasibility Cluster Randomised Controlled Intervention Trial in Schools. *Prevention Science* 2018; **19**: 1079–90.

3 Kirby D. Effective approaches to reducing adolescent unprotected sex, pregnancy, and childbearing. *Journal of Sex Research* 2002; **39**: 51–7.

4 Kirby D in I. Mathtech Questionnaires (Sexuality Questionnaires for Adolescents). In: Fisher TD Davis CM YWLDSL, ed. Handbook of sexuality-related measures, 3rd edn. London: Routledge, 2011: 35–47.

5 Pleck JH, Sonenstein FL, Ku LC. Masculinity Ideology: Its Impact on Adolescent Males' Heterosexual Relationships. *Journal of Social Issues* 1993; **49**: 11–29.

6 Quinn-Nilas C, Milhausen RR, Breuer R, *et al.* Validation of the Sexual Communication Self-Efficacy Scale. *Health Education and Behavior* 2015; **43**: 165–71.

7 Lohan M, Aventin Á, Maguire L, *et al.* Increasing boys' and girls' intentions to avoid teenage pregnancy: a cluster randomised controlled feasibility trial of an interactive video drama-based intervention in post-primary schools in Northern Ireland. *Public Health Research* 2017; **5**: 1–344.

8 Craig P, Dieppe P, Macintyre S, Michie S, Nazareth I, Petticrew M. Developing and evaluating complex interventions: the new Medical Research Council guidance. *BMJ* 2008; **337**.

9 May C. Towards a general theory of implementation. *Implement Sci* 2013; **8**: 18.

10 Moore GF, Audrey S, Barker M, *et al.* Process evaluation of complex interventions: Medical Research Council guidance. *BMJ* 2015; **350**: h1258.

11 Braun V, Clarke V. Using thematic analysis in psychology. *Qualitative Research in Psychology* 2006; **3**: 77–101.

12 Lincoln YS, Guba E. Naturalistic Inquiry. Newbury Park, CA: Sage Publications, 1985.

13 UK Government. Policy statement: Relationships Education, Relationships and Sex Education, and Personal, Social Health and Economic Education. 2017.

14 Welsh Government. The Future of the Sex and Relationships Education Curriculum in Wales Recommendations of the Sex and Relationships Education Expert Panel. 2017.

15 Council for the Curriculum Examinations and Assessment. Relationships and Sexuality Education Guidance An Update for Post-Primary Schools. Belfast, 2019.

16 The Scottish Government. Conduct of Relationships, Sexual Health and Parenthood Education in Schools. 2014.

17 National Children's Bureau for the Sex Education Forum. 2020.

18 Aventin Á, Gough A, McShane T, *et al.* Engaging parents in digital sexual and reproductive health education: Evidence from the JACK Trial. *Reproductive Health* 2020; **17**. https://reproductive-health-journal.biomedcentral.com/articles/10.1186/s12978-020-00975-y.

19 Office for National Statistics. Earning and hours worked, occupation by four-digit SOC: ASHE table 14 (2017 revised). Earning and hours worked, occupation by four-digit SOC: ASHE table 14 (accessed Dec 13, 2019).

20 Curtis L, Burns A. Unit Costs of Health and Social Care 2019. 2019.

21 NICE. Guide to the methods of technology appraisal 2008. 2008.

22 Boots. 2019. https://www.boots.com/ (accessed July 20, 2021).

23 Nherera L, P. Jacklin. A model to assess the cost-effectiveness of Sex and Relationship Education (SRE) developed for NICE public health guidance on personal, social, health and economic (PSHE) education. 2009.

24 Wang LY, Davis M, Robin L, Collins J, Coyle K, Baumler E. Economic Evaluation of Safer Choices A School-Based Human Immunodeficiency Virus, Other Sexually Transmitted Diseases, and Pregnancy Prevention Program. *Arch Pediatr Adolesc Med* 2000; **154**: 1017–24.

25 Public Health England. Economic Analysis Estimation of the Return on Investment (ROI) for publicly funded contraception in England. 2018 https://assets.publishing.service.gov.uk/government/uploads/system/uploads/attachment_data/file/730292/contraception_return_on_investment_report.pdf (accessed July 2, 2021).

26 Wellings K, Jones KG, Mercer CH, *et al.* The prevalence of unplanned pregnancy and associated factors in Britain: findings from the third National Survey of Sexual Attitudes and Lifestyles (Natsal-3). *The Lancet* 2013; **382**. DOI:10.1016/S0140-6736(13)62071-1.

27 Henderson M, Ecob R, Wight D, Abraham C. What explains between-school differences in rates of smoking? *BMC Public Health* 2008; **8**. DOI:10.1186/1471-2458-8-218.

28 Brooks F, Magnusson J, Klemera E, Chester K, Spencer N, Smeeton N. HBSC England National Report: Health behaviour in school-aged children (HBSC). 2015.

29 Roberts C. HBSC Wales National Report: Health behaviour in school-aged children (HBSC): key findings. 2014.

30 Currie C, Levin K, Todd J. HBSC Scotland National Report: Health behaviour in school-aged children (HBSC). 2014.

31 Schubotz D. Messed up? Sexual lifestyles of 16 year olds in Northern Ireland. 2011.

32 Office for National Statistics. Conceptions in England and Wales: 2017. 2017. https://www.ons.gov.uk/peoplepopulationandcommunity/birthsdeathsandmarriages/conceptionandfertilityrates/bulletins/conceptionstatistics/2017.

33 Office for National Statistics. Abortion statistics for England and Wales. 2018. https://www.gov.uk/government/statistics/abortion-statistics-for-england-and-wales.

34 HES Online. Statistics. 2009. http://www.hesonline.nhs.uk/Ease/servlet/ContentServer?siteID=1937&categoryID=1064.

35 Public Health Scotland. Maternity and Births. 2017. https://www.isdscotland.org/Health-Topics/Maternity-and-Births/Births/.

36 NISRA. Births (administrative geographies) 1999-2014. Northern Ireland Statistics & Research Agency (NISRA), 2015.

37 Office for National Statistics. Population estimates. 2017. https://www.ons.gov.uk/peoplepopulationandcommunity/populationandmigration/populationestimates.

38 Health Protection Agency. Diagnoses and rates of selected STIs seen at UK GUM clinics by country and age group: 2002–2006. 2007. https://www.gov.uk/government/organisations/health-protection-agency.

39 Lewis J, White PJ. Changes in chlamydia prevalence and duration of infection estimated from testing and diagnosis rates in England: a model-based analysis using surveillance data, 2000-15. *Lancet Public Health* 2018; **3**: e271–e278.

40 Sonnenberg P, Clifton S, Beddows S, Field N, Soldan K, Tanton C. Prevalence, risk factors, and uptake of interventions for sexually transmitted infections in Britain: findings from the National Surveys of Sexual Attitudes and Lifestyles (Natsal). *The Lancet* 2013; **382**: 1795–806.

41 Turner KM, Adams EJ, LaMontagne DS, Emmett L, Baster K, Edmunds WJ. Modelling the effectiveness of chlamydia screening in England. *Sex Transm Infect* 2006; **82**: 496–502.

42 Low N, McCarthy A, Macleod J, Salisbury C, Campbell R, Roberts TE. Epidemiological, social, diagnostic and economic evaluation of population screening for genital chlamydial infection. *Health Technol Assess* 2007; **11**.

43 Chlamydia Advisory Group on behalf of the National Chlamydia Screening Steering Group. National Chlamydia Screening Programme. England, 2004.

44 Town K, Furegato M, Field N, Hughes G. Estimating gonorrhoea prevalence in young heterosexual men and women attending community-based sexual health services to inform decisions on gonorrhoea testing. *Epidemiol Infect* 2017; **145**: 1682–7.

45 Brunham R, Plummer F. A general model of sexually transmitted disease epidemiology and its implications for control. *The Medical Clinics of North America* 1990; **75**: 1339–52.

46 Rothenberg R, Potterat JJ, Koplan JP. The algebra of condoms and abstinence. *Sex Transm Dis* 2005; **32**: 252–4.

47 Howell-Jones R. Baseline HPV Epidemiology Studies. 2008. www.cornwall.nhsuk/CornishMicrobiologicalSociety/Presentations/PDF/HPVEpidemiologyRHowellJones.pdf.

48 Weller SC, Davis-Beaty K. Condom effectiveness in reducing heterosexual HIV transmission. *Cochrane Database of Systematic Reviews* 2002; **2012**. DOI:10.1002/14651858.CD003255.

49 Schwarz EB, Smith R, Steinauer J, Reeves MF, Caughey AB. Measuring the Effects of Unintended Pregnancy on Women's Quality of Life. *Contraception* 2008; **78**: 204–10.

50 Goldhaber-Fiebert J, Brandeau M. Evaluating Cost-Effectiveness of Interventions that Affect Fertility and Childbearing: How Health Effects are Measured Matters. *Med Decis Making* 2015; **35**: 818–46.

51 Insinga RP, Dasbach EJ, Myers ER. The health and economic burden of genital warts in a set of private health plans in the United States. *Clin Infect Dis* 2003; **36**: 1397–403.

52 Maclean CC, Stringer JS. Potential cost-effectiveness of maternal and infant antiretroviral interventions to prevent mother-to-child transmission during breast-feeding. *J Acquir Immune Defic Syndr* 2005; **38**: 570–7.

53 Hu D, Hook EW, Goldie SJ. Screening for Chlamydia trachomatis in women 15 to 29 years of age: a cost-effectiveness analysis. *Ann Intern Med* 2004; **141**: 501–13.

54 Reference Costs, NHS Trust Cost Schedules. 2017. http://www.dh.gov.uk/prod_consum_dh/groups/dh_digitalassets/documents/digitalasset/dh_098948.xls.

55 Belfield D, Crawford C, Sibieta L. Long-run comparisons of spending per pupil across different stages of education. *I F Stud* 2017.

56 Licchetta M, Stelmach M. Fiscal sustainability analytical paper: Fiscal sustainability and public spending on health. *O B Responsib* 2016.

57 Nherera L, Jacklin P. A model to assess the cost-effectiveness of Sex and Relationship Education (SRE) developed for NICE public health guidance on personal, social and health education (PSHE). NICE, 2009.

58 Wellings K, Wilkinson P, Gundy C. Teenage pregnancy strategy evaluation: final report. 2005.

59 Office for National Statistics. Earning and hours worked, occupation by four-digit SOC: ASHE table 14. .

60 Lucas S. Unprotected Nation: the financial and economic impacts of restricted contraceptive and sexual health services. *D Economics* 2013.

61 Chandra A, Martinez GM, Mosher WD, Abma JC, Jones J. Fertility, family planning, and reproductive health of U.S. women: data from the 2002 National Survey of Family Growth. *Vital Health Stat 23* 2005; **25**: 1–160.

62 Montouchet C, Trussell J. Unintended pregnancies in England in 2010: costs to the National Health Service (NHS). *Contraception* 2013; **87**: 149–53.

63 Earnshaw J, Gavin L. NICE guide to the methods of technology appraisal. 2008.
